# Treatment Recommendations for Clinical Deterioration on the Wards: Development and Validation of Machine Learning Models

**DOI:** 10.2196/81642

**Published:** 2026-01-16

**Authors:** Eric Pulick, Kyle A Carey, Tonela Qyli, Madeline K Oguss, Jamila K Picart, Leena Penumalee, Lily K Nezirova, Sean T Tully, Emily R Gilbert, Nirav S Shah, Urmila Ravichandran, Majid Afshar, Dana P Edelson, Yonatan Mintz, Matthew M Churpek

**Affiliations:** 1 Department of Industrial and Systems Engineering, University of Wisconsin-Madison, Madison, WI, United States; 2 Department of Medicine, University of Chicago, Chicago, IL, United States; 3 Department of Medicine, University of Wisconsin-Madison, 610 Walnut Street, Suite 515, Madison, WI, 53726, United States, 1 6082629564; 4 Department of Surgery, University of Michigan, Ann Arbor, MI, United States; 5 Department of Medicine, Northwestern Memorial Hospital, Chicago, IL, United States; 6 Department of Medicine, Loyola University Medical Center, Chicago, IL, United States; 7 Department of Medicine, Endeavor Health, Evanston, IL, United States; 8 Department of Data Analytics, Endeavor Health, Evanston, IL, United States; 9 Department of Biostatistics and Medical Informatics, University of Wisconsin-Madison, Madison, WI, United States

**Keywords:** clinical deterioration, critical illness, early warning score, hospital rapid response team, machine learning, artificial intelligence, chart review, clinical decision support system

## Abstract

**Background:**

Clinical deterioration in general ward patients is associated with increased morbidity and mortality. Early and appropriate treatments can improve outcomes for such patients. While machine learning (ML) tools have proven successful in the early identification of clinical deterioration risk, little work has explored their effectiveness in providing data-driven treatment recommendations to clinicians for high-risk patients.

**Objective:**

This study established ML performance benchmarks for predicting the need for 10 common clinical deterioration interventions. This study also compared the performance of various ML models to inform which types of approaches are well-suited to these prediction tasks.

**Methods:**

We relied on a chart-reviewed, multicenter dataset of general ward patients experiencing clinical deterioration (n=2480 encounters), who were identified as high risk using a Food and Drug Administration–cleared early warning score (electronic Cardiac Arrest Risk Triage score). Manual chart review labeled each encounter with gold-standard lifesaving treatment labels. We trained elastic net logistic regression, gradient boosted machines, long short-term memory, and stacking ensemble models to predict the need for 10 common deterioration interventions at the time of the deterioration elevated risk score. Models were trained on encounters from 3 health systems and externally validated on encounters from a fourth health system. Discriminative performance, assessed by the area under the receiver operating characteristic curve (AUROC), was the primary evaluation metric.

**Results:**

Discriminative performance varied widely by model and prediction task, with AUROCs typically ranging from 0.7 to 0.9. Across all models, antiarrhythmics were the easiest treatment to predict (mean AUROC 0.866, SD 0.012) while anticoagulants were the hardest to predict (mean AUROC 0.660, SD 0.065). While no individual modeling approach outperformed the others across all tasks, the gradient boosted machines tended to show the best individual performance. Additionally, the stacking ensemble, which combined predictions from all models, typically matched or outperformed the best-performing individual model for each task. We also demonstrated that a sizable fraction of patients in our evaluation cohort were untreated at the time of the deterioration elevated risk score, highlighting an opportunity to leverage ML tools to decrease treatment latency.

**Conclusions:**

We found variability in the discrimination of ML models across tasks and model approaches for predicting lifesaving treatments in patients with clinical deterioration. Overall performance was high, and these models could be paired with early warning scores to provide clinicians with timely and actionable treatment recommendations to improve patient care.

## Introduction

### Background

Previous studies have demonstrated that clinical deterioration on the hospital wards is associated with increased morbidity and mortality [1-5]. Deteriorating ward patients who require intensive care unit (ICU) transfer account for a disproportionate fraction of ICU deaths, with their mortality rates exceeding those of patients admitted directly to the ICU [3,6]. Early and appropriate interventions are associated with improved outcomes for patients experiencing acute physiological deterioration [7-11]. Despite this knowledge, delays in care are common and associated with increased mortality [12-16], motivating the development of new approaches to improve care for this high-risk population.

Efforts to improve interventions for patients with clinical deterioration can be divided into 2 domains: *identification* (ie, earlier detection of high-risk patients) and *response* (ie, the actions taken to address deterioration), also called the afferent and efferent limbs of a rapid response system [17]. Much existing work has focused on identification, as earlier detection of high-risk patients naturally supports earlier interventions; we refer to the study by Mann et al [18] for a recent survey of approaches related to early warning scores for early identification. Broadly, these approaches identify physiological changes, such as changes in vital signs, that tend to precede deterioration [5,19,20]. Methodologies vary widely, although much recent work has focused on integrating advanced machine learning (ML) approaches with electronic health records (EHRs) to process risk scores automatically [19,21,22]. Single- and multicenter studies implementing these types of early warning systems have demonstrated promising improvements to patient outcomes [21,23-25].

Despite the progress in the identification arm of the system, there is far less work analyzing how automated ML approaches can be applied to similarly improve the response arm. Identification is a necessary condition for *initiating* treatment, but it is not sufficient to ensure that a patient receives the *most appropriate* treatment (or treatments) in a timely fashion. This is particularly important because early warning systems often focus on nonspecific deterioration risk instead of monitoring for a specific syndrome like sepsis [26]. Ideally, early warning systems that flag high-risk patients would additionally supply data-driven treatment recommendations. A recommendation could serve as a clinical decision support tool, either to reinforce clinician intuition or to prompt treatments that the clinician might not have initially considered. Clinicians using similar artificial intelligence (AI) clinical decision support tools in related fields have been shown to outperform both the supporting AI model and clinician judgment individually (eg, in pathology [27] and radiology [28]).

However, treatment recommendation ML models that are tied to clinical deterioration early warning scores have not yet been developed, in large part, because such models cannot be properly trained on EHR data without significant additional clinician input. Although EHR data can provide information regarding what treatments a patient received, expert manual chart review is required to assess which treatments they received (or did not receive) were appropriate and directed at the underlying cause of deterioration. Without chart review, models can only learn to mimic the status quo, rather than provide gold-standard treatment recommendations. Chart-reviewed datasets of this kind are rare and typically limited in size or to single centers [29]. As a result, it is currently unclear what level of performance clinicians can expect from treatment recommendation algorithms for general ward clinical deterioration. Furthermore, it is unknown which types of ML modeling approaches will perform best in this context.

### Contribution

In this study, we train a collection of ML models to predict lifesaving treatments for general ward patients with clinical deterioration. These models are designed to supplement a generalized early warning system by providing treatment suggestions for clinician decision support. We rely on a large, multicenter dataset with gold-standard treatment recommendations established by manual chart review [30]. These models set benchmark performance standards for different types of treatment recommendations, and we discuss the advantages and disadvantages of the various ML model types under study.

## Methods

### Study Cohort

We used a study cohort built from 4000 chart-reviewed patient encounters, originally introduced by Churpek et al [30]; we reiterate key aspects of the cohort’s construction here.

Encounters were sampled from 4 health systems: University of Chicago Medicine, the University of Wisconsin-Madison Hospital, the Loyola University Medical Center, and 4 hospitals within Endeavor Health. These samples were drawn from the population of each health system’s encounters that met the inclusion criteria established in Textbox 1. Collectively, the encounters occurred between 2007 and 2020.

Textbox 1. **All encounters satisfying the following criteria at the 4 noted health systems were eligible to be sampled as part of the study cohort.**

- The patient was at least 18 years of age;
- Clinician provider notes (eg, admission history and physical discharge summary) were available for the encounter;
- During their encounter, the patient was admitted to the hospital and spent time on a medical-surgical (non–intensive care unit) ward.

Across the 4 health systems, 919,319 encounters met the inclusion criteria. EHR data associated with these eligible encounters were evaluated using the electronic Cardiac Arrest Risk Triage score (eCART), an early warning score that uses demographics, vital signs, and laboratory results to predict clinical deterioration (ie, cardiac arrest, ICU transfer, or death) [19]. Among these eligible encounters, 91,131 included 1 or more instances where the eCART model met the threshold for elevated risk of clinical deterioration (top 5% risk score) while on the medical-surgical (non-ICU) ward. For brevity, we refer to this event as an elevated risk score. From each health system, 1000 encounters with at least 1 elevated risk score were randomly sampled for manual chart review (4000 total) by expert acute care physicians. In this work, 5 of the encounters from the University of Wisconsin-Madison Hospital were ultimately excluded due to a lack of EHR data availability, leaving 995 encounters for that health system and 3995 overall. These 3995 encounters were further filtered by chart review to the final cohort size of 2480 based on the presence of a true deterioration event (ie, an occurrence of clinical deterioration rather than a false alarm) during or following the elevated risk score. A complete flow diagram is provided in Multimedia Appendix 1.

### Ethical Considerations

The study was approved by the institutional review board (IRB) at each health system with a waiver of informed consent. IRB approval was given under University of Chicago Biological Sciences Division IRB #18‐0447, University of Wisconsin-Madison IRB #2019‐1258, Loyola University Medical Center IRB #215437, and Endeavor Health IRB #11‐0539. All direct identifiers were deidentified before analysis to ensure privacy and confidentiality. Participants did not receive compensation for this data analysis, as this was a retrospective analysis and no direct contact with participants occurred.

### Measures

#### Patient Measurements

A complete list of patient measurements included in our modeling is provided in Multimedia Appendix 2. Approximately 50 measurement types were included in our modeling. These measurements included demographic information (eg, age and sex), vital signs (eg, heart rate and temperature), and laboratory measurements (eg, electrolytes and blood cell counts). These measurement types were selected by expert consensus as ubiquitous measures available in the EHR. The measurements were used to construct features for the different algorithms used in our modeling. Refer to the “Feature Engineering” section for further description of the features used in our tested models.

The EHR also included information about the treatments each patient received. However, this treatment information was not included as an input to our models and was only used for evaluation purposes (ie, to assess when or whether a patient received a treatment deemed lifesaving by manual chart review). We chose to exclude treatments from the model features to prevent label leakage into the dataset. Not only could leakage artificially inflate assessed model performance, but inclusion would have also allowed current treatment practices to influence model predictions.

#### Chart Review

The 1000 encounters sampled from each health system were manually chart-reviewed by trained reviewers in each health system. The complete chart review procedures are described in the study by Churpek et al [30]; we reiterate relevant procedures and outcomes in this study.

First, the reviewers assessed whether the elevated risk score corresponded to a true deterioration event or to a false alarm (eg, due to a spike in heart rate associated with the patient getting out of bed). Of the 3995 encounters in the cohort, 2480 included 1 or more true deterioration events. For these cases, the reviewers recorded 1 or more treatments that would ultimately be considered lifesaving for the patient’s deterioration event. For encounters that contained more than 1 deterioration event, the chart review assessed the first such event. The reviewers used information from both before and after the elevated risk score (eg, clinician notes following treatment initiation that describe diagnostic test results and the response to therapy). Lifesaving treatments included both drug interventions, such as antiarrhythmics or steroids, and nondrug interventions, such as transfusions or ventilation. Additionally, while nearly all patients received the treatment (or treatments) indicated to be lifesaving by the reviewers at some point in their encounter, the chart-reviewed treatment was not limited to the treatments the patient received. For instance, if a patient died before the treatment could be administered, it was still included as a lifesaving treatment during chart review. The chart review process *did not* assign an optimal time for initiating each labeled intervention. As our goal was to evaluate the performance of ML treatment recommendation algorithms at the time of an elevated risk score, all chart-reviewed labels were chosen such that they would have been appropriate to administer at the time of the elevated risk score.

### Labels and Prediction Tasks

For each encounter, the chart review process established 1 or more treatments to be lifesaving for the patient. These treatments served as the labels for our predictive modeling. We considered the 10 most common treatments indicated by chart review, given in Textbox 2. Thus, the prediction problem was to predict a patient’s need for each of the 10 treatment categories using the patient’s EHR measurements (processed appropriately into model features) at the time of the patient’s elevated risk score. This approach poses the problem as *multilabel* prediction (ie, prediction for each treatment type occurs in parallel) as opposed to *multiclass* prediction (ie, treating combinations of treatments as possible labels with only 1 label assigned to each encounter).

Textbox 2. **Treatment labels in descending order of prevalence across encounters at the Loyola University Medical Center (n=622 encounters). Encounters at this health system were used as our test set, while encounters from the remaining 3 health systems were used for model training and validation. For each treatment, we parenthetically note the number of positive-labeled cases. A single encounter may be labeled with multiple lifesaving treatments, so positive labels do not sum to the total number of encounters.**

- Antimicrobial (including antibiotics, antifungals, and antivirals; n=300)
- Fluid bolus (n=231)
- Antiarrhythmic (including beta-blockers and AV nodal blocking agents, n=111)
- Diuretic (n=93)
- Inhaled bronchodilator (including nebulizer treatments and asthma medications, n=79)
- Transfusion (n=60)
- Invasive ventilator (n=53)
- Vasoactive (including inotropes, n=49)
- Anticoagulant (n=36)
- Steroid (n=29)

### Tested Models

#### Model Types

A primary goal of our modeling was to assess whether certain model types showed better or worse discriminative performance on different treatment prediction tasks. As such, we trained traditional, non–deep learning prediction models, namely elastic net logistic regression (LR) and gradient boosted machines, as well as deep learning time-series models, specifically a type of recurrent neural network called a long short-term memory (LSTM) model [31]. LR was implemented using Scikit-learn [32], gradient boosted machines were implemented using tree-based Extreme Gradient Boosting, referred to as XGB [33], and LSTMs were implemented using PyTorch [34]. Both single- and multilabel LSTMs were evaluated for the various prediction tasks. In the single-label case, we trained unique LSTMs (including hyperparameter tuning) for each prediction task; this mirrors the process for LR and XGB, which also natively consider only a single label per model. In the multilabel case, we trained 1 LSTM model (ie, with 1 set of hyperparameters) that simultaneously made predictions for all 10 treatment prediction tasks.

Given the success of ensemble learning approaches in numerous health care prediction tasks [35,36], we also evaluated the performance of a stacking ensemble learner in this treatment recommendation context. Stacking, sometimes also called late fusion, involves training a meta-learner from the outputs of individual models, effectively learning appropriate weighting values to assign to predictions made by each model [37,38]. In this study, we used an elastic net LR meta-learner, trained using the prediction probabilities from the individual model as features.

#### Feature Engineering

While features for each model type relied on the same set of EHR measurements, structural differences in the models necessitated different approaches in feature engineering. We provide an overview of these differences here and refer readers to a complete list of features and construction procedures for each model in Multimedia Appendix 2.

The largest difference in features between LR or XGB and the LSTM models was their handling of temporal information. LR and XGB do not directly process time-series data and thus required the creation of a single set of features to describe each encounter. The first portion of these LR and XGB features was the last available value for each EHR measurement type at the time of the elevated risk score. XGB can handle missing feature values (eg, for an individual with no available measurements of a certain value before the elevated risk score), so XGB models were trained with a featurization of the dataset that preserved missingness. LR cannot accommodate missing measurements, so we created a separate version of the features for LR that imputed missing values with medians from the training set; these LR and XGB feature sets were otherwise identical. In addition to the last-available measurement values, we also included a set of temporal summary statistics for certain measurement types over the 24 hours preceding the elevated risk score (eg, minimum or maximum values, means and SDs, and rates of change over given time intervals). These quantities allowed for the encoding of near-term temporal information about the patient and have been shown to improve the performance of these models in previous work on early warning scores [20].

In contrast, LSTM models are designed to handle sequences of temporal measurements for each encounter. For the LSTMs, we resampled the raw time-series data to uniform intervals. The interval length (2, 4, or 6 h) was a tunable hyperparameter for each of the prediction tasks (ie, treatment types). Regardless of the interval length, a last-value-pulled-forward approach was used for resampling the value at each time step. If no value was available during the resampling interval, it was pulled forward from the previous resampled value. If no previous resampled value was available, it was imputed using the median value from the training set. Resampling was performed relative to the time of the elevated risk score, meaning the elevated risk score time was used as the anchor point and the EHR data were processed backward from that time using the specified interval length. In addition to the resampled measurement values, a tunable hyperparameter in our LSTM models was the use of imputation Booleans (ie, features that take the value 1 when a given measurement is imputed and 0 when there is a true measurement) [39]. This allowed the LSTM to also learn patterns associated with missing versus true measurement values.

For all models except XGB, we used the minimum and maximum observed values in the training set to rescale features to the interval [0,1]. The minimum and maximum values used for rescaling were specific to the features constructed for each model (ie, values in the resampled time-series features for the LSTM were only used for rescaling the LSTM features, not the LR features, and vice versa). XGB is scale-independent, so variable scaling was not performed for these models.

#### Model Training

Data from encounters containing deterioration events at 3 of the health systems (University of Chicago Medicine, 483 encounters; University of Wisconsin-Madison Hospital, 656 encounters; and Endeavor Health, 719 encounters) were combined to form a training set (1858 encounters). The 622 encounters in the fourth health system, Loyola University Medical Center, were used as a held-out test set. Structural differences between LR and XGB, the LSTMs, and the stacking ensemble required slightly different tuning and training procedures and are described further in this study.

LR and XGB followed a common fitting procedure aside from LR’s use of features with imputation and XGB’s use of features with missingness. Both LR and XGB models had tunable hyperparameters (eg, regularization method for LR and number of boosting rounds for XGB) that were evaluated with cross-validation. Specifically, the best-performing hyperparameters were established by grid search during 4 repeats of 3-fold stratified cross-validation of the training set. The area under the receiver operating characteristic curve (AUROC) was used as the cross-validation scoring metric [40]. A complete set of hyperparameter ranges and chosen hyperparameters for LR and XGB is provided in Multimedia Appendix 2 and Multimedia Appendix 3. After identifying the best-performing hyperparameters via grid search, the LR and XGB models were retrained on the complete training set and evaluated on the test set.

Training differed slightly for the LSTMs as we used early stopping as a form of model regularization; this meant that the number of passes through the training set was included in hyperparameter tuning. Specifically, in each iteration of the same repeated 3-fold stratified cross-validation process, 2 folds of the training set were used for model training, and 1 was used for validation. After each pass through the training data, we evaluated the trained model’s AUROC on the validation encounters. We repeated this process until 10 epochs passed without the current epoch’s validation AUROC exceeding the best-observed validation AUROC across past epochs. The best-observed AUROC was used as the scoring value for that iteration of cross-validation. We recorded the epoch count associated with the best-observed validation AUROC and took the mean of this value across the 12 total iterations of repeated cross-validation to obtain the tuned epoch value for that set of hyperparameters.

In addition to tuning common hyperparameters, such as the learning rate or number of hidden units, we explored the effect of various other LSTM structures from the literature. For instance, we included a target replication parameter that allowed for intermediate predictions (ie, those that would be made at time steps before the elevated risk score) to also factor into the model’s loss function [36,41]. We also included the option to use channel-wise inputs as a binary hyperparameter [42]; when this option was used, each measurement channel was passed through an additional LSTM with its own tunable hyperparameters. This allowed models to capture specific trends in individual measurement streams before these quantities were combined in the primary LSTM model. As with LR and XGB, a complete set of hyperparameter ranges and chosen values is available in Multimedia Appendix 2 and Multimedia Appendix 3. As LSTM training was more time-consuming than that of LR and XGB, we used Optuna (Preferred Networks) [43], a package that uses Bayesian optimization to efficiently sample candidate hyperparameters, to coordinate LSTM hyperparameter tuning rather than performing a grid search. As with LR and XGB, after identifying the best-performing hyperparameters, we retrained a model on the complete training set and evaluated its performance on the test set.

As our stacking ensemble model was an elastic net LR model, it followed the same hyperparameter tuning process as our individual LR models. However, special care was needed to construct the training dataset for this stacking model. Recall that a stacking model uses weighted predictions from each submodel (eg, XGB) to make its predictions. To learn an appropriate weighting, a stacking model must be trained with out-of-sample predictions from each submodel. For each of the submodels, we iteratively reconstructed a complete set of the training encounters with out-of-sample predictions; the training set was divided into 5 folds, and each submodel type was trained on 4 of the folds (using the best performing hyperparameters identified previously) to produce out-of-sample predictions for the remaining fold. Iterating across all 5 folds allowed for complete reconstruction of the training set with out-of-sample predictions. We repeated this process 5 times to produce a dataset suitable for repeated cross-validation (5 repeats of 5-fold cross-validation). We used these out-of-sample predictions to perform hyperparameter tuning and identify the best-performing hyperparameters for the stacking models. We then trained the final stacking model using the best-performing hyperparameters on the complete set of out-of-sample predictions and evaluated the stacking model’s performance on the test set.

#### Evaluation Criteria

Our primary evaluation criterion for the different models was discriminatory ability, assessed using the AUROC. We express uncertainty in calculated AUROC values using nonparametric bootstrapped 95% CIs [44]. As a secondary metric, we assessed model calibration using calibration curves and Brier scores, with uncertainties also expressed using bootstrapped 95% CIs. Finally, Shapley Additive Explanations (SHAP) values were used to provide model explainability results, as described in the study by Lundberg and Lee [45].

## Results

### Demographic Information

Table 1 provides a summary of the demographic characteristics of the study cohort, including separation by encounters at the train and test sites. The test site encounters came from a separate health system but show similar demographic characteristics to the encounters from the 3 health systems used to form the training set.

**Table 1.** Demographic information for patient populations at the studied health systems.

Measure	All sites	Train sites	Test site
Encounter count, n (%)	2480 (100)	1858 (74.9)	622 (25.1)
Age (y), median (IQR)	70 (50-84)	71 (59-86)	67 (57-79)
Female, n (%)	1244 (50.1)	944 (50.8)	300 (48.2)
Black, n (%)	492 (19.8)	359 (19.3)	133 (21.3)
Elevated risk score value (eCART^a^ score), median (IQR)	50 (40-76)	51 (40-78)	49 (40-69)
Length of stay before elevated risk score (hr), median (IQR)	20.3 (6.4-67.2)	18.4 (5.7-60.5)	28.2 (8.1-88.8)
Length of stay after elevated risk score (hr), median (IQR)	126.9 (68.1-241.0)	124.4 (67.0-234.8)	133.7 (72.0-260.7)
In ICU^b^ before elevated risk score, n (%)	453 (18.2)	327 (17.5)	126 (20.2)
In operating room before elevated risk score, n (%)	401 (16.1)	285 (15.3)	116 (18.6)
ICU transfer after elevated risk score, n (%)	721 (29)	501 (26.9)	220 (35.3)
In-hospital mortality, n (%)	357 (14.3)	253 (13.6)	104 (16.7)

a eCART: electronic Cardiac Arrest Risk Triage.

b ICU: intensive care unit.

### Timing of Treatment Initiation

To give insight into the treatment initiation practices observed in the test site encounters, Table 2 summarizes the fraction of patients who received each treatment during particular time periods in their encounter. Additional information regarding treatment initiation timing can be found in Multimedia Appendix 2. While most patients received the lifesaving treatments assessed by chart review at some point in their encounter, a sizable fraction of patients were untreated at the time of the elevated risk score. This untreated patient fraction varied by treatment, ranging from roughly 20% in the case of antiarrhythmics to nearly 90% in the case of vasoactives.

**Table 2.** Timing of lifesaving treatments for the 622 test set encounters from the Loyola University Medical Center. Each row describes summaries of treatment initiation timing for the encounters labeled as requiring that treatment during chart review.

Treatment	Encounters, n (%)	^a^Patient received treatment during specified time window, n (%)				
		Any time during encounter	±48 h of ERS^b^	Any time before ERS	<48 h before ERS	<24 h before ERS
Antimicrobial	300 (48.2)	295 (98.3)	291 (97)	217 (72.3)	202 (67.3)	129 (43)
Fluid bolus	231 (37.1)	185 (80.1)	171 (74)	107 (46.3)	89 (38.5)	73 (31.6)
Antiarrhythmic	111 (17.8)	110 (99.1)	108 (97.3)	87 (78.4)	85 (76.6)	39 (35.1)
Diuretic	93 (14.9)	92 (98.9)	85 (91.4)	56 (60.2)	53 (57)	30 (32.2)
Inhaled bronchodilator	79 (12.7)	76 (96.2)	75 (94.9)	58 (73.4)	58 (73.4)	26 (32.9)
Transfusion	60 (9.6)	50 (83.3)	47 (78.3)	23 (38.3)	18 (30)	15 (25)
Vasoactive	49 (7.9)	46 (93.9)	35 (71.4)	6 (12.2)	4 (8.2)	1 (2)
Anticoagulant	36 (5.8)	34 (94.4)	33 (91.7)	25 (69.4)	23 (63.9)	12 (33.3)
Steroid	29 (4.7)	28 (96.6)	25 (86.2)	14 (48.3)	14 (48.3)	5 (17.2)

a Subcolumns indicate if a patient was treated during the noted period of their encounter (ie, each subcolumn gives a particular summary of treatment practices for the encounters labeled as needing the treatment described in each row). For instance, chart review labeled 300 of the 622 encounters with antimicrobials as a lifesaving treatment. Among these 300 patient encounters, 295 (98.3%) received antimicrobial treatment at some point during their encounter, 291 (97%) received antimicrobial treatment specifically within ±48 hours of their elevated risk score, and so on. Patients may have received treatment multiple times during their encounter. Note that the chart review process considered patients on invasive, mechanical ventilation to be intensive care unit patients, thus making them ineligible for chart review sampling and implying that no patients in this cohort were receiving invasive ventilation at the time of the elevated risk score. The electronic health record did not have a reliable signal indicating when invasive ventilation began, so we do not report treatment summaries for this treatment.

b ERS: elevated risk score.

### Model Performance

**Figure 1.**
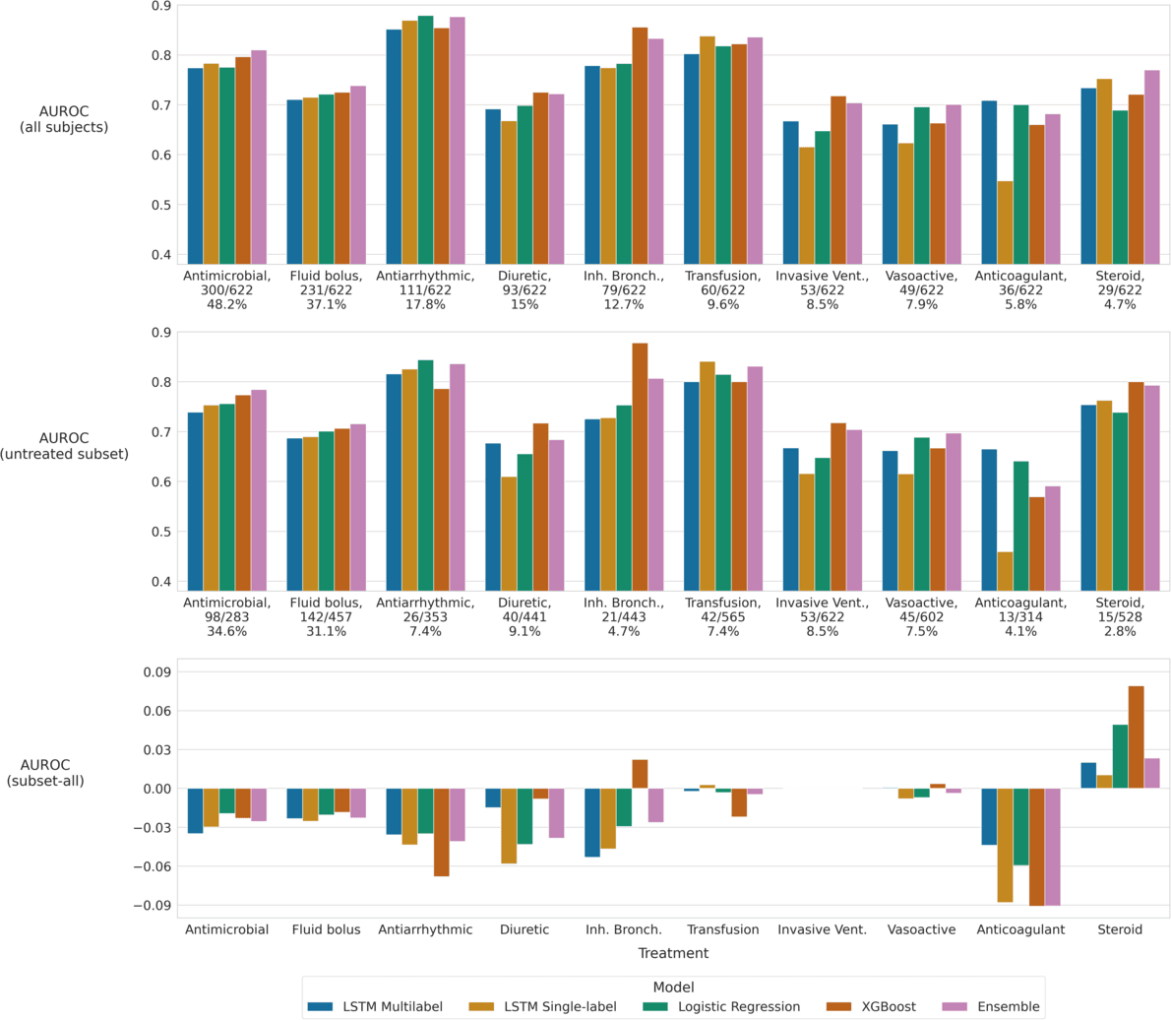
Area under the receiver operating characteristic curve (AUROC) performance for each model type on the 10 treatment prediction tasks (given as bar plots). The top panel summarizes model performance when evaluated on all patients in the test cohort. The middle panel summarizes model performance when evaluated only on subjects in the test cohort who were not receiving the designated treatment at the time of the elevated risk score (determined based on whether the patient received the treatment within the 48 h before the elevated risk score). Below each label in the top and middle panels is the ratio of positive cases to total cases. The bottom panel summarizes the difference in AUROC values between the first and second panels (AUROC on the untreated subset minus AUROC on all subjects). Negative values denote a decrease in performance on the untreated subset compared with the full test cohort. In all panels, models are grouped together for each treatment. Inh. Bronch.: inhaled bronchodilator; LSTM: long short-term memory; Vent.: ventilator; XGBoost: Extreme Gradient Boosting.

Figure 1 summarizes the discriminative performance of the tested models across the 10 prediction tasks, assessed by AUROC; this summary includes a separation of performance on the complete test cohort from performance on the subset of patients not actively receiving treatment at the time of the elevated risk score. Model performance varied widely by model type and prediction task, with AUROCs typically ranging from 0.7 to 0.9.

Table 3 summarizes the discriminative performance of the models for each prediction task, specifically by averaging the AUROC performance across model types for each treatment. Mean AUROC values are presented for both the complete test site cohort and the subset of test site patients not actively receiving treatment at the time of the elevated risk score. The rank-ordering of mean AUROCs varied slightly between these cohorts. Broadly, the models tended to perform best on prediction for antiarrhythmics, transfusions, and inhaled bronchodilators and performed worst on anticoagulants, vasoactive agents, and invasive ventilation.

**Table 3.** Summary of model performance for the different treatment types. Performance is assessed by the mean area under the receiver operating characteristic curve performance of all model types (including the stacking ensemble) for each treatment. Mean values are calculated for both the full test cohort and the untreated patient subset.

Treatment	Full test set (AUROC^a^), mean (SD)	Untreated subset (AUROC), mean (SD)
Antiarrhythmic	0.866 (0.013)	0.822 (0.022)
Transfusion	0.823 (0.014)	0.818 (0.018)
Inhaled bronchodilator	0.805 (0.037)	0.778 (0.065)
Antimicrobial	0.788 (0.015)	0.761 (0.018)
Steroid	0.733 (0.031)	0.770 (0.026)
Fluid Bolus	0.722 (0.011)	0.700 (0.012)
Diuretic	0.701 (0.024)	0.669 (0.040)
Invasive ventilator	0.671 (0.042)	0.671 (0.042)
Vasoactive	0.669 (0.031)	0.666 (0.032)
Anticoagulant	0.660 (0.065)	0.585 (0.080)

a AUROC: area under the receiver operating characteristic curve.

Table 4 summarizes the relative performance of the different model types. Specifically, model performance was assessed by weighted AUROC performance across tasks and the mean AUROC rank ordering of the models. Results are presented for both the complete test site cohort and the subset of test site encounters not actively receiving treatment at the time of the elevated risk score. While no individual model universally outperformed the others, XGB showed the best weighted AUROC across tasks. The stacking ensemble offered improved performance over the individual models, typically matching or exceeding the AUROC of the best performing individual model and showing the best overall weighted AUROC performance.

**Table 4.** Summary of tested model performance. Weighted model AUROCs^a^, mean AUROC model rank across all algorithms, and mean AUROC model rank among individual models are provided for both patient populations (ie, the complete test site cohort and the untreated patient subset). Weighted AUROCs were calculated using the number of positive cases for each prediction task as a weighting factor. Mean AUROC ranking calculations weighted each prediction task equally. SD values are noted in parentheses.

Model	Performance on full test set			Performance on untreated subset		
	Weighted AUROC	Mean AUROC Rank	Mean AUROC Rank (individual only)	Weighted AUROC	Mean AUROC Rank	Mean AUROC Rank (individual only)
Ensemble	0.781	1.7 (0.64)	—^b^	0.743	1.8 (0.60)	—
XGB^c^	0.769	2.5 (1.20)	1.8 (0.87)	0.737	2.4 (1.43)	1.8 (1.08)
LR^d^	0.755	3.1 (1.14)	2.3 (0.90)	0.720	3.0 (1.10)	2.2 (0.87)
Multilabel LSTM^e^	0.749	3.9 (1.22)	3.0 (1.00)	0.712	3.9 (1.22)	3.0 (1.00)
Single-label LSTM	0.744	3.8 (1.40)	2.9 (1.22)	0.699	3.9 (1.22)	3.0 (1.00)

a AUROC: area under the receiver operating characteristic curve.

b Not applicable.

c XGB: Extreme Gradient Boosting.

d LR: logistic regression.

e LSTM: long short-term memory.

**Figure 2.**
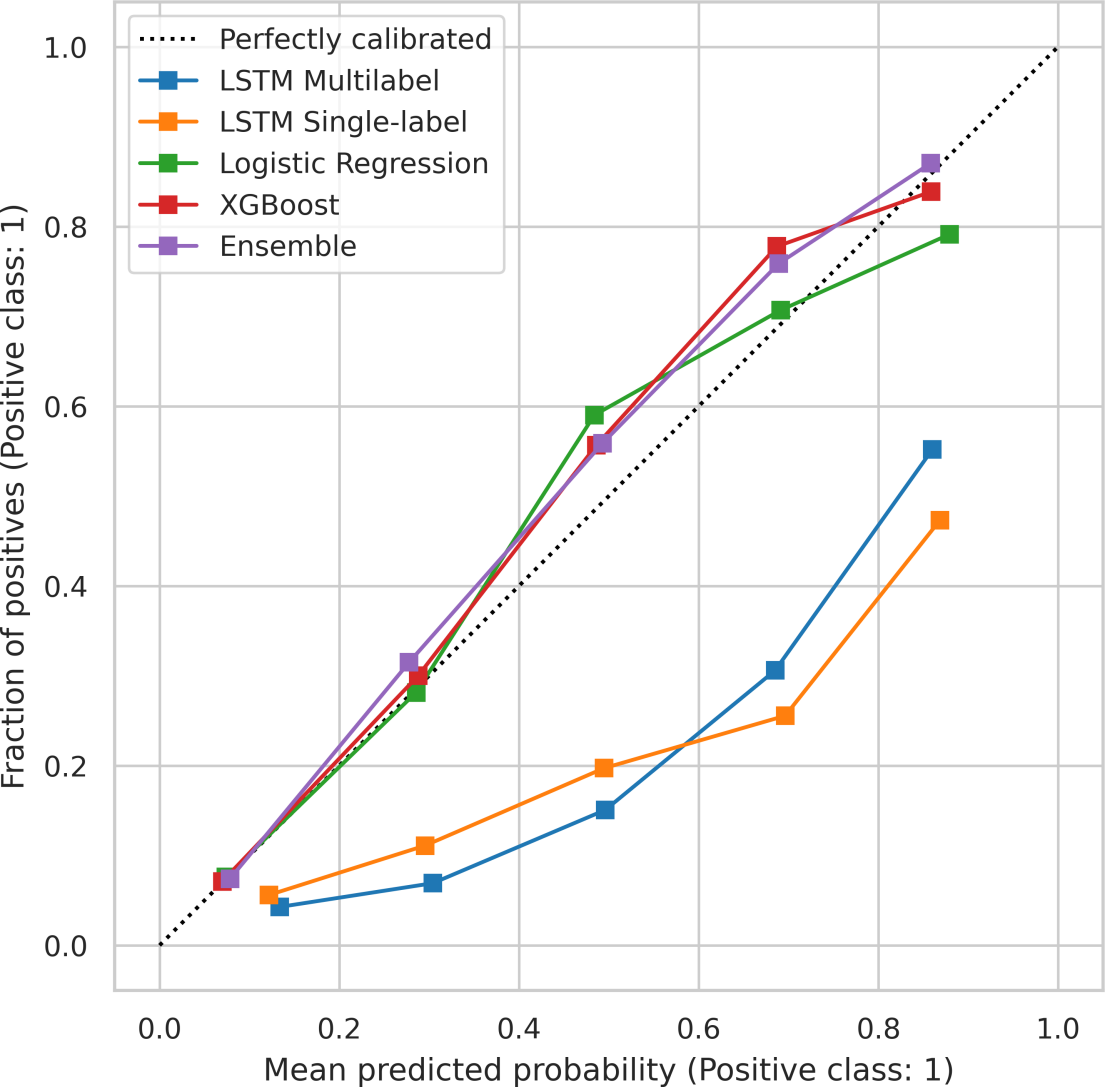
Calibration curves for the tested algorithms using a pooled set of predictions (ie, all prediction tasks are combined). Pooling was performed as many individual tasks had too few positive cases to create meaningful calibration curves. LSTM: long short-term memory; XGBoost: Extreme Gradient Boosting.

Figure 2 shows calibration curves for each modeling approach when pooling predictions across all tasks. To further assess calibration performance, we provide Brier scores and task-specific calibration curves in Multimedia Appendix 2. We note that we did not apply calibration postprocessing techniques and instead evaluated the intrinsic calibration of the methods. The classical ML approaches tended to be well-calibrated, while the LSTMs showed poor calibration. Global feature importance plots, assessed using SHAP values, for the 3 most common treatments (antimicrobials, fluid boluses, and antiarrhythmics) as well as case evaluations of steroid and anticoagulant prediction can be found in the “Feature Importance” section in Multimedia Appendix 2.

## Discussion

### Principal Findings

In this multicenter study developing and comparing treatment recommendation algorithms for high-risk hospitalized patients, we found that predictive performance varied significantly by model type and prediction task, typically with AUROC values of 0.7‐0.9. When assessed by the mean AUROC of all models, including the ensemble, we observed the highest discriminative performance on antiarrhythmic, transfusion, and bronchodilator prediction (mean AUROCs 0.866 [SD 0.012], 0.823 [SD 0.014], and 0.805 [SD 0.037], respectively) and observed the lowest discriminative performance on invasive ventilation, vasoactive agents, and anticoagulant prediction (mean AUROCs 0.671 [SD 0.042], 0.669 [SD 0.031], and 0.660 [SD 0.065], respectively). Overall, the XGB algorithm was the best-performing individual model type, and an ensemble of all model types further improved performance. However, each individual model had the best discriminative performance in at least 1 prediction task. The timing of treatment initiation in the test site cohort varied by therapy, and the models generally performed similarly for patients receiving and not receiving therapy at the time of the elevated risk score (except for anticoagulants). To the best of our knowledge, these are the first models in the published literature that use gold-standard chart-reviewed training data to make treatment predictions at the time of a clinical deterioration elevated risk score. These models could be incorporated alongside early warning scores to enhance clinical decision-making and prompt earlier, lifesaving treatments.

### Predictive Performance by Treatment Type

We first examine the differences in performance across the various treatment types, which ranged in AUROC values from approximately 0.7 to 0.9. Antiarrhythmic prediction had the highest AUROC, while anticoagulant prediction had the lowest AUROC, although several additional treatment types showed similar performance to anticoagulant prediction (diuretics, invasive ventilation, and vasoactives all had mean AUROCs close to 0.7). A potential contributor to differences in performance is likely the presence (or absence) of useful predictive signals in the structured EHR data used in this study. For instance, changes in heart rate, which is a structured data element included in our models, often signal a patient’s need for antiarrhythmics, while treatments like diuretics rely on additional measurement modalities that we did not include (eg, chest X-ray images or physical observations showing signs of fluid overload). To validate this intuition, we performed SHAP analysis for the 3 most common treatment types (antimicrobials, fluid boluses, and antiarrhythmics; Multimedia Appendix 2). We found that the most important features for each treatment type agreed well with clinical intuition. For instance, the most important features for antimicrobial prediction related to temperature, white blood cell counts, heart rate, and lactate levels. The most important features for fluid bolus prediction related to blood pressure, electrolytes, and heart rate. For antiarrhythmic prediction, features related to heart rate were the most important.

As a result, we would expect our models to perform best when the most relevant patient attributes for a given treatment type are contained within structured EHR data, such as vital signs and laboratory values. This naturally motivates the fusion of additional data sources for such models in the future to enhance their discriminative performance. Fusion methods, particularly intermediate and late fusion approaches, have been shown to improve predictive performance by integrating additional modalities, such as imaging and clinical notes [46,47]. In particular, mixture-of-experts frameworks show promise for effectively handling multimodal data even when certain modalities are missing [48]. More broadly, however, it may be the case that certain conditions are easier to predict than others, for instance, due to lower variability in patient presentation. Even without additional input modalities, the models presented here can be helpful sources of recommendations, especially if clinicians are informed about which treatment recommendations are most accurate.

### Predictive Performance by Model Type

We found that no single algorithm uniformly outperformed the others on all prediction tasks, and the rank-ordering of algorithm performance varied across tasks. The absence of a universally superior algorithm is consistent with recent literature comparing baseline models (eg, LR) with gradient boosted trees (eg, XGB) and deep learning approaches [49]. Broadly, however, XGB showed the best individual model performance, with the highest weighted AUROC across tasks and the best mean AUROC ranking among individual algorithms. This also agrees with recent literature demonstrating that boosted decision trees tend to outperform other modeling approaches in prediction tasks for moderately sized tabular datasets [49-51]. Importantly, however, all tested models were the best-performing *individual* model in at least 1 prediction type; XGB performed best on 5 tasks, LR performed best on 2 tasks, the single-label LSTMs performed best on 2 tasks, and the multilabel LSTM performed best on 1 task. Additionally, while discrimination was our primary evaluation metric, model calibration is also an important consideration for use in a medical recommendation setting, as it measures whether predicted probabilities reflect observed outcome frequencies [52]. This work evaluated the calibration performance of the tested methods without applying any corrective postprocessing techniques. XGB and LR both showed good calibration, while the LSTM approaches were poorly calibrated. We suspect that this is partially due to the use of class weighting for the LSTMs on all tasks to reduce the computational burden of hyperparameter tuning. Furthermore, modern neural networks are known to exhibit poor calibration [53]. Numerous methods are available to improve model calibration via postprocessing, such as Platt scaling or isotonic regression [53-55]. However, best practices for these techniques rely on the use of additional held-out data, so model types with better intrinsic calibration may be especially valuable in this data-scarce setting.

Given the varied performance of the individual models, it was not surprising to see that the stacking ensemble, which incorporated weighted contributions from each individual model, tended to outperform the individual models. Specifically, the ensemble had the best overall performance on 4 individual prediction tasks, had the highest weighted AUROC across tasks, and had a higher mean AUROC ranking across prediction tasks when ranked alongside individual models. The ensemble also tended to show the best calibration across the tested methods. This performance is consistent with existing literature, which has shown the benefits of ensemble approaches in medical diagnostics [35]. However, we note that the AUROC improvement of the ensemble over that of the individual models was typically modest. Therefore, clinician stakeholders will need to assess whether the improved performance merits additional implementation or explainability costs compared with implementing a single model like XGB. Hyperparameter tuning for the LSTM approaches, for instance, carried significantly more computational burden compared with LR and XGB. Furthermore, while we focused on establishing the discriminative performance of the different methods, future work will need to consider the misclassification costs associated with each of the treatment types to guide such modeling decisions.

### Timing of Treatment Initiation

The timing of treatment initiation for chart-reviewed lifesaving treatments varied by treatment type. We focused on the fraction of individuals receiving lifesaving treatment within 48 hours before their elevated risk score, as these treatments are more likely to be clinician responses to the deterioration event of interest. For some interventions (eg, antimicrobials, antiarrhythmics, bronchodilators, and anticoagulants), approximately 75% of patients received the corresponding treatment before the elevated risk score. However, for other interventions (eg, steroids, fluid boluses, transfusions, and vasoactive medications), fewer than half of the encounters had their treatment initiated before the elevated risk score. As a result, we expect that our models will offer different benefits to some patients and conditions compared with others. For instance, in some cases, the models reinforce clinician intuition (ie, where treatment has already been initiated), whereas in others, they would prompt treatment initiation. Furthermore, nearly all patients received the lifesaving treatment at some point during their encounter, with slight variations by treatment type. As noted previously, the early initiation of appropriate treatments is associated with improved patient outcomes [7-11], reinforcing the potential benefit of AI decision support tools in recommending lifesaving treatments.

### Predictive Performance for Untreated Patients

We further assessed predictive performance for the subset of test site patients who were not receiving each treatment type at the time of the elevated risk score. A patient was included in this untreated subset if they did not receive the noted treatment within the 48 hours before the elevated risk score for the deterioration event under study. We noted a small but near-universal drop in AUROC across algorithms and treatment types, typically less than 0.05. The effect is more pronounced for some treatment types than others. There are several possible explanations for this phenomenon. Cases with near-negligible differences, such as vasoactive medications, are likely explained by near-identical study populations (ie, AUROC calculations are performed on nearly the same population since very few patients receive such a treatment). Beyond these cases, we expect that some of the performance drop is explained by the nature of this patient subset; by excluding patients who already received treatment from clinicians, we are presumably left with a cohort that is more difficult to correctly diagnose and treat. Thus, we would expect the algorithms to perform worse when evaluated specifically on this more challenging patient subset. A further contributor to the drop in AUROC is likely the presence of label leakage through certain EHR measurements. Because some treatments have clear markers in the EHR, once initiated, it is possible for the models to use these quantities for shortcut learning [56]. Anticoagulants showed the largest drop in performance between the overall and subset cohorts and serve as a particularly salient example for this effect; heparin treatment impacts a patient’s partial thromboplastin time, which is one of the measurements used by the models. If the trained model learns to rely on the presence of treatment proxies to perform prediction, the absence of this signal in the untreated cohort is likely to result in poorer performance. Finally, we note that steroid prediction showed a counterintuitive increase in performance across all models when measured on the untreated patient subset. SHAP analysis of relevant model features cross-referenced with differences in these patient populations did not suggest a clear explanation for this increase in discriminative performance (Multimedia Appendix 2). We suspect that this is an artifact of steroids having the most severe class imbalance, making random variation in positive cases more pronounced than the other prediction tasks.

### Case Study of Anticoagulant Prediction

We highlight the prediction of anticoagulants to illustrate 2 important performance trends for the studied algorithms. First, anticoagulant prediction was a task with noticeably better performance by the multilabel LSTM compared with XGB. While XGB tended to outperform the LSTM approaches in general, here we see the potential value of time-series models relative to non–time-series approaches for certain prediction tasks. As LR and XGB are not fundamentally time-series methods, these approaches required hand-crafted temporal features to capture such information (eg, SD of a measurement type over the previous day). SHAP analysis of the XGB model’s test set predictions (Multimedia Appendix 2) suggested that the 3 most important covariates for anticoagulant prediction were temporal summary statistics (in descending order): SD of heart rate over the past 24 hours, slope of temperature measurements over the past 24 hours, and SD of temperature measurements over the past 24 hours. Furthermore, for the most important feature, 24-hour SD of heart rate, higher values were associated with the positive class, suggesting that these patients experienced significant variation in heart rate measurements. Thus, it may be that the better performance observed from the multilabel LSTM owes to its ability to learn relevant patterns directly from the time-series data for these measurements rather than relying on less informative temporal summary statistics.

However, anticoagulants also highlight a possible pitfall of using data-hungry, deep learning approaches in this relatively low-data regime [57]; while the multilabel LSTM had the best performance for this treatment type, the single-label LSTM had the worst performance. Direct consideration of time-series data may allow higher capacity models to extract additional information for prediction, but it may also lead to poorer performance through overfitting, even with the types of regularization used in the training of our models. This is especially relevant for the single-label LSTMs, where we performed dedicated hyperparameter tuning for each prediction task. To this end, we observed that single-label LSTMs tended to outperform multilabel LSTMs for prediction tasks with less class imbalance, and vice versa.

### Deployment Considerations

While our primary focus in this study is to establish predictive performance benchmarks for various ML approaches, we also discuss several important points related to the real-world deployment of these models. Foremost, we envision these models providing suggestions to clinicians to enhance their decision-making, rather than having decision-making authority themselves. However, even in this recommender capacity, several relevant implementation considerations follow.

The first consideration is the predictive performance of such models with respect to novel populations and to subpopulations. While the results presented in this manuscript come from model validation on an external site, all 4 sites included in this study are regionally similar, academic health systems in the United States. Further study will be required to evaluate model predictive performance in other settings, such as community or international hospitals, where varying degrees of data shift may meaningfully impact performance [58]. Even in settings with significant data shifts, models like those trained in this manuscript may provide a valuable foundation for transfer learning using setting-specific data [59,60]. Furthermore, additional study is needed to evaluate the performance of such models on particular patient subpopulations to assess concerns related to algorithmic fairness [61,62].

Second, the prospective operation of these models relies on a minimum level of in-hospital data infrastructure, including the real-time availability of structured EHR data and the ability to calculate the model scores [63]. While this infrastructure is readily available in the academic medical centers described in this manuscript, this may not be true in community or international hospital settings. Furthermore, we briefly noted how these recommendation models could be augmented to include other measurement modalities, such as imaging or clinical notes; these modalities are expected to improve model performance but may not be available for real-time prediction models in some hospitals, leading to wider differences in performance between high- and low-resource hospital settings.

Third, much additional study is needed to evaluate the most effective integration of these treatment suggestions into clinical workflows. While these models are naturally tied to the usage of clinical deterioration early warning systems, such as eCART, there is significant flexibility in how treatment recommendations are actually delivered to clinicians (eg, the use of thresholding vs probability scores, the integration into other rapid response system elements, or the temporal and visual manner of delivery). The field of human factors provides a principled means to design effective clinical decision support system implementations in close collaboration with relevant stakeholders [64-66]. These efforts will be a critical component of future work in order to address common problems with early-warning–type systems, such as alarm fatigue and cognitive overload.

Finally, we briefly address broader ethical concerns with clinical decision support systems in medical decision-making, such as those related to misclassification and clinician reliance. An important finding from this study was the difference in predictive performance across treatment categories and model types. One aspect of future work will be assessing false positive and false negative costs associated with each treatment type to inform tradeoffs when presenting threshold-based model scores. As incorrect treatment initiation costs may vary significantly across treatments, the level of clinically meaningful model performance is expected to differ by treatment type. These types of considerations may motivate the use of more complex models for certain treatment types but not others, even if doing so incurs greater certification costs or effort. With respect to clinician reliance, we emphasize that such models can never be expected to be perfectly accurate and that, in this proposed framework, the clinician has ultimate responsibility for choosing whether to initiate treatment.

### Limitations

We emphasize and reiterate some limitations of our study. First, our work does not show that a treatment recommendation algorithm improves outcomes for patients, such as decreasing treatment latency and time in the hospital. This needs to be assessed through future prospective implementation studies to determine if the use of our recommendation algorithms improves patient care. We expect that prospective implementation efforts will raise important human factors considerations (eg, trust between a clinician and the clinical decision support) that we do not address in this work. Next, while this is a large, chart-reviewed dataset, it is still relatively small compared with datasets typically used to train medical ML models. This is an especially important consideration for the performance of the deep learning (ie, LSTM) approaches, as the dataset may be too small to fully leverage the additional signal present in each encounter’s time series data. Furthermore, while we used a multicenter dataset, the included sites are all regionally similar health systems in the Midwest region of the United States; additional sites would be needed to assess how well these results generalize to other health systems. Finally, our work focuses primarily on the discriminative and calibration performance of the tested models; we do not address concrete tradeoffs for initiation or incorrect initiation of the different treatment types. We plan to incorporate these factors into future work, as they help inform tradeoffs in modeling decisions and enable a more complete evaluation of algorithm performance.

### Conclusion

This work provides benchmark discrimination and calibration performance for a variety of ML methods on a collection of common treatment recommendation tasks. The difficulty of the recommendation tasks was found to vary widely by treatment, with mean model AUROCs ranging from approximately 0.7 (eg, anticoagulants or vasoactives) to nearly 0.9 (eg, antiarrhythmics). While no individual model uniformly outperformed all other models across prediction tasks, XGB had the best weighted discriminative performance across tasks and exhibited well-calibrated predictions. An ensemble combining both classical ML and time-series, deep learning approaches tended to match or outperform the best-performing individual model in each prediction task in both discrimination and calibration. The observed performance suggests that such ML tools may serve as valuable clinical decision support in tandem with generalized early warning scores to improve the timely and appropriate treatment of deteriorating general ward patients.

## Supplementary material

10.2196/81642 Multimedia Appendix 1 Patient flow diagram for this study.

10.2196/81642 Multimedia Appendix 2 Primary technical appendix.

10.2196/81642 Multimedia Appendix 3 Supplementary technical appendix, specifically containing oversized tables requiring landscape orientation.

10.2196/81642 Checklist 1 Machine learning reporting checklist, following the Consolidated Reporting Guidelines for Prognostic and Diagnostic Machine Learning Modeling Studies (CREMLS) format.

## Abbreviations

AI: artificial intelligence
AUROC: area under the receiver operating characteristic curve
eCART: electronic Cardiac Arrest Risk Triage score
EHR: electronic health record
ICU: intensive care unit
IRB: institutional review board
LR: logistic regression
LSTM: long short-term memory
ML: machine learning
SHAP: Shapley Additive Explanations
XGB: Extreme Gradient Boosting

## References

[1] Jones D, Mitchell I, Hillman K, Story D (2013). Defining clinical deterioration. Resuscitation.

[2] Churpek MM, Wendlandt B, Zadravecz FJ, Adhikari R, Winslow C, Edelson DP (2016). Association between intensive care unit transfer delay and hospital mortality: a multicenter investigation. J Hosp Med.

[3] Liu V, Kipnis P, Rizk NW, Escobar GJ (2012). Adverse outcomes associated with delayed intensive care unit transfers in an integrated healthcare system. J Hosp Med.

[4] Chen J, Bellomo R, Flabouris A, Hillman K, Assareh H, Ou L (2015). Delayed emergency team calls and associated hospital mortality: a multicenter study. Crit Care Med.

[5] Delgado MK, Liu V, Pines JM, Kipnis P, Gardner MN, Escobar GJ (2013). Risk factors for unplanned transfer to intensive care within 24 hours of admission from the emergency department in an integrated healthcare system. J Hosp Med.

[6] Escobar GJ, Greene JD, Gardner MN, Marelich GP, Quick B, Kipnis P (2011). Intra-hospital transfers to a higher level of care: contribution to total hospital and intensive care unit (ICU) mortality and length of stay (LOS). J Hosp Med.

[7] Boersma E, Maas AC, Deckers JW, Simoons ML (1996). Early thrombolytic treatment in acute myocardial infarction: reappraisal of the golden hour. The Lancet.

[8] Rivers E, Nguyen B, Havstad S (2001). Early goal-directed therapy in the treatment of severe sepsis and septic shock. N Engl J Med.

[9] Plant P, Owen J, Elliott M (2000). Early use of non-invasive ventilation for acute exacerbations of chronic obstructive pulmonary disease on general respiratory wards: a multicentre randomised controlled trial. The Lancet.

[10] Liu VX, Fielding-Singh V, Greene JD (2017). The timing of early antibiotics and hospital mortality in sepsis. Am J Respir Crit Care Med.

[11] Hodgetts TJ, Brett A, Castle N (1998). The early management of meningococcal disease. Emerg Med J.

[12] Young MP, Gooder VJ, McBride K, James B, Fisher ES (2003). Inpatient transfers to the intensive care unit. J Gen Intern Med.

[13] Ray P, Birolleau S, Lefort Y (2006). Acute respiratory failure in the elderly: etiology, emergency diagnosis and prognosis. Crit Care.

[14] Cowie MR, Anker SD, Cleland JGF (2014). Improving care for patients with acute heart failure: before, during and after hospitalization. ESC Heart Fail.

[15] Lachkhem Y, Rican S, Minvielle É (2018). Understanding delays in acute stroke care: a systematic review of reviews. Eur J Public Health.

[16] Han X, Spicer A, Carey KA (2021). Identifying high-risk subphenotypes and associated harms from delayed antibiotic orders and delivery. Crit Care Med.

[17] DeVita MA (2024). Textbook of Rapid Response Systems: Concept and Implementation.

[18] Mann KD, Good NM, Fatehi F (2021). Predicting patient deterioration: a review of tools in the digital hospital setting. J Med Internet Res.

[19] Churpek MM, Yuen TC, Winslow C (2014). Multicenter development and validation of a risk stratification tool for ward patients. Am J Respir Crit Care Med.

[20] Churpek MM, Adhikari R, Edelson DP (2016). The value of vital sign trends for detecting clinical deterioration on the wards. Resuscitation.

[21] Escobar GJ, Liu VX, Schuler A, Lawson B, Greene JD, Kipnis P (2020). Automated identification of adults at risk for in-hospital clinical deterioration. N Engl J Med.

[22] Churpek MM, Carey KA, Snyder A (2024). Multicenter development and prospective validation of eCARTv5: a gradient boosted machine learning early warning score. medRxiv.

[23] Winslow CJ, Edelson DP, Churpek MM (2022). The impact of a machine learning early warning score on hospital mortality: a multicenter clinical intervention trial. Crit Care Med.

[24] McDonnell A, Tod A, Bray K, Bainbridge D, Adsetts D, Walters S (2013). A before and after study assessing the impact of a new model for recognizing and responding to early signs of deterioration in an acute hospital. J Adv Nurs.

[25] Kollef MH, Chen Y, Heard K (2014). A randomized trial of real-time automated clinical deterioration alerts sent to a rapid response team. J Hosp Med.

[26] Escobar GJ, Dellinger RP (2016). Early detection, prevention, and mitigation of critical illness outside intensive care settings. J Hosp Med.

[27] Bulten W, Balkenhol M, Belinga JJA (2021). Artificial intelligence assistance significantly improves Gleason grading of prostate biopsies by pathologists. Mod Pathol.

[28] Rodríguez-Ruiz A, Krupinski E, Mordang JJ (2019). Detection of breast cancer with mammography: effect of an artificial intelligence support system. Radiology.

[29] Blackwell JN, Keim-Malpass J, Clark MT (2020). Early detection of in-patient deterioration: one prediction model does not fit all. Crit Care Explor.

[30] Churpek MM, Ingebritsen R, Carey KA (2024). Causes, diagnostic testing, and treatments related to clinical deterioration events among high-risk ward patients. Crit Care Explor.

[31] Hochreiter S, Schmidhuber J (1997). Long short-term memory. Neural Comput.

[32] Pedregosa F, Varoquaux G, Gramfort A (2011). Scikit-learn: machine learning in Python. J Mach Learn Res.

[33] Chen T, Guestrin C XGBoost: a scalable tree boosting system.

[34] Paszke A, Gross S, Massa F PyTorch: an imperative style, high-performance deep learning library. https://dl.acm.org/doi/10.5555/3454287.3455008.

[35] Mahajan P, Uddin S, Hajati F, Moni MA (2023). Ensemble learning for disease prediction: a review. Healthcare (Basel).

[36] Lipton ZC, Kale DC, Elkan C, Wetzel R (2015). Learning to diagnose with LSTM recurrent neural networks. arXiv.

[37] Hastie T, Tibshirani R, Friedman J (2009). The Elements of Statistical Learning: Data Mining, Inference, and Prediction, Second Edition.

[38] Wolpert DH (1992). Stacked generalization. Neural Netw.

[39] Lipton ZC, Kale DC, Wetzel R (2016). Modeling missing data in clinical time series with RNNs. JMLR W&CP.

[40] Hanley JA, McNeil BJ (1982). The meaning and use of the area under a receiver operating characteristic (ROC) curve. Radiology.

[41] Lee CY, Xie S, Gallagher P, Zhang Z, Tu Z Deeply-supervised nets.

[42] Harutyunyan H, Khachatrian H, Kale DC, Ver Steeg G, Galstyan A (2019). Multitask learning and benchmarking with clinical time series data. Sci Data.

[43] Akiba T, Sano S, Yanase T, Ohta T, Koyama M Optuna: a next-generation hyperparameter optimization framework.

[44] Carpenter J, Bithell J (2000). Bootstrap confidence intervals: when, which, what? A practical guide for medical statisticians. Stat Med.

[45] Lundberg SM, Lee SI A unified approach to interpreting model predictions. https://proceedings.neurips.cc/paper_files/paper/2017/file/8a20a8621978632d76c43dfd28b67767-Paper.pdf.

[46] Soenksen LR, Ma Y, Zeng C (2022). Integrated multimodal artificial intelligence framework for healthcare applications. NPJ Digit Med.

[47] Huang SC, Pareek A, Seyyedi S, Banerjee I, Lungren MP (2020). Fusion of medical imaging and electronic health records using deep learning: a systematic review and implementation guidelines. NPJ Digit Med.

[48] Han X, Harris C, Ho N, Nguyen H, FuseMoE SS (2024). Mixture-of-Experts Transformers for Fleximodal Fusion Adv Neural Inf Process Syst 37.

[49] McElfresh D, Khandagale S, Valverde J When do neural nets outperform boosted trees on tabular data?. https://dl.acm.org/doi/10.5555/3666122.3669459.

[50] Grinsztajn L, Oyallon E, Varoquaux G Why do tree-based models still outperform deep learning on typical tabular data?. https://dl.acm.org/doi/10.5555/3600270.3600307.

[51] Shwartz-Ziv R, Armon A (2022). Tabular data: deep learning is not all you need. Information Fusion.

[52] Alba AC, Agoritsas T, Walsh M (2017). Discrimination and calibration of clinical prediction models: users’ guides to the medical literature. JAMA.

[53] Guo C, Pleiss G, Sun Y, Weinberger KQ On calibration of modern neural networks. https://dl.acm.org/doi/10.5555/3305381.3305518.

[54] Platt J (1999). Probabilistic outputs for support vector machines and comparisons to regularized likelihood methods. Adv Large Margin Classif.

[55] Zadrozny B, Elkan C Transforming classifier scores into accurate multiclass probability estimates. https://dl.acm.org/doi/proceedings/10.1145/775047.

[56] Geirhos R, Jacobsen JH, Michaelis C (2020). Shortcut learning in deep neural networks. Nat Mach Intell.

[57] van der Ploeg T, Austin PC, Steyerberg EW (2014). Modern modelling techniques are data hungry: a simulation study for predicting dichotomous endpoints. BMC Med Res Methodol.

[58] Subasri V, Krishnan A, Kore A (2025). Detecting and remediating harmful data shifts for the responsible deployment of clinical AI models. JAMA Netw Open.

[59] Weiss K, Khoshgoftaar TM, Wang D (2016). A survey of transfer learning. J Big Data.

[60] Desautels T, Calvert J, Hoffman J (2017). Using transfer learning for improved mortality prediction in a data-scarce hospital setting. Biomed Inform Insights.

[61] Olfat M, Mintz Y Flexible regularization approaches for fairness in deep learning. https://ieeexplore.ieee.org/xpl/mostRecentIssue.jsp?punumber=9303728.

[62] Dobbe R, Krendl Gilbert T, Mintz Y (2021). Hard choices in artificial intelligence. Artif Intell.

[63] Gebler R, Reinecke I, Sedlmayr M, Goldammer M (2025). Enhancing clinical data infrastructure for AI research: comparative evaluation of data management architectures. J Med Internet Res.

[64] Carayon P, Hoonakker P, Hundt AS (2020). Application of human factors to improve usability of clinical decision support for diagnostic decision-making: a scenario-based simulation study. BMJ Qual Saf.

[65] Hekman DJ, Barton HJ, Maru AP (2024). Dashboarding to monitor machine-learning-based clinical decision support interventions. Appl Clin Inform.

[66] Barton HJ, Maru A, Leaf MA (2024). Academic detailing as a health information technology implementation method: supporting the design and implementation of an emergency department-based clinical decision support tool to prevent future falls. JMIR Hum Factors.

[67] (2025). SMPH (Public) / Department of Medicine / UW-ICU-Data-Science-Lab-Public / cd_treatment_recommendation. GitLab.

[68] Klement W, El Emam K (2023). Consolidated reporting guidelines for prognostic and diagnostic machine learning modeling studies: development and validation. J Med Internet Res.

