# Supplementary material for: Treatment Recommendations for Clinical Deterioration on the Wards: Development and Validation of Machine Learning Models
Source: JMIR AI. 2026 Jan 16;5:e81642. doi: 10.2196/81642 (PMC12810948; doi:10.2196/81642)
Supplement: Multimedia Appendix 1 [file ai-v5-e81642-s001.pptx]

## Slide 1
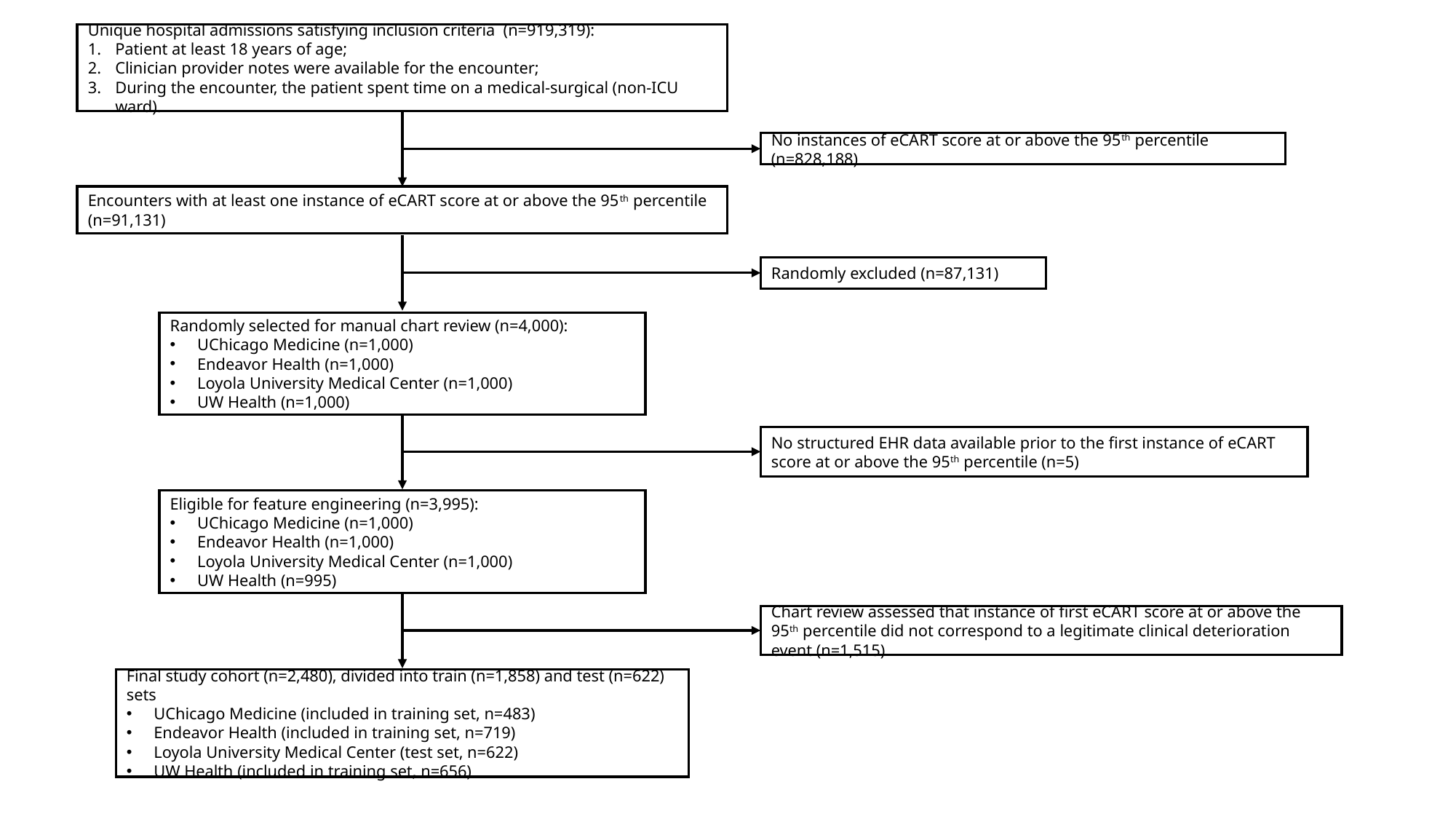

Unique hospital admissions satisfying inclusion criteria (n=919,319):
Patient at least 18 years of age;
Clinician provider notes were available for the encounter;
During the encounter, the patient spent time on a medical-surgical (non-ICU ward).
No instances of eCART score at or above the 95th percentile (n=828,188)
Encounters with at least one instance of eCART score at or above the 95th percentile (n=91,131)
Randomly excluded (n=87,131)
Randomly selected for manual chart review (n=4,000):
UChicago Medicine (n=1,000)
Endeavor Health (n=1,000)
Loyola University Medical Center (n=1,000)
UW Health (n=1,000)
No structured EHR data available prior to the first instance of eCART score at or above the 95th percentile (n=5)
Eligible for feature engineering (n=3,995):
UChicago Medicine (n=1,000)
Endeavor Health (n=1,000)
Loyola University Medical Center (n=1,000)
UW Health (n=995)
Chart review assessed that instance of first eCART score at or above the 95th percentile did not correspond to a legitimate clinical deterioration event (n=1,515)
Final study cohort (n=2,480), divided into train (n=1,858) and test (n=622) sets
UChicago Medicine (included in training set, n=483)
Endeavor Health (included in training set, n=719)
Loyola University Medical Center (test set, n=622)
UW Health (included in training set, n=656)
