# Supplementary material for: Treatment Recommendations for Clinical Deterioration on the Wards: Development and Validation of Machine Learning Models
Source: JMIR AI. 2026 Jan 16;5:e81642. doi: 10.2196/81642 (PMC12810948; doi:10.2196/81642)
Supplement: Multimedia Appendix 2 [file ai-v5-e81642-s002.docx]

Multimedia Appendix 1 Table of Contents

[Multimedia Appendix 1 2](#_Toc214564128)

[Measurement Types 2](#_Toc214564129)

[Model Features 2](#_Toc214564130)

[Logistic Regression & Extreme Gradient Boosting 2](#_Toc214564131)

[Long Short-Term Memory 3](#_Toc214564132)

[Model Structure and Hyperparameters 3](#_Toc214564133)

[Logistic Regression 3](#_Toc214564134)

[Extreme Gradient Boosting 4](#_Toc214564135)

[Long Short-Term Memory 4](#_Toc214564136)

[Initial Validation Procedures 4](#_Toc214564137)

[Final Hyperparameter Ranges 5](#_Toc214564138)

[Best Performing Hyperparameters 6](#_Toc214564139)

[Treatment Timing 6](#_Toc214564140)

[Calibration Curves 7](#_Toc214564141)

[Performance Metric Confidence Intervals 8](#_Toc214564142)

[Area under the ROC Curve 8](#_Toc214564143)

[Brier Scores 9](#_Toc214564144)

[Feature Importance 10](#_Toc214564145)

[SHAP Analysis of Steroid Treatments 10](#_Toc214564146)

[SHAP Analysis of Anticoagulant Treatments 12](#_Toc214564147)

[Additional SHAP Analysis for Common Treatments 12](#_Toc214564148)

[References 15](#_Toc214564149)

# Multimedia Appendix 1

This appendix serves as the primary technical appendix for the main text. Tables large enough to require landscape orientation can be found in Multimedia Appendix 2.

## Measurement Types

Table 1 describes the various measurement channels used for feature engineering. These measurement channels were selected by expert review of available signals across the 4 studied health systems’ electronic health records (EHR) data. In most cases, these measurement values were used directly as model features, but in some cases, they required minor post-processing (e.g. creating trend quantities). These processes are defined in a subsequent section describing the features used in each model (see Section ‘Model Features’).

**Table 1.** Measurements used for the construction of model features, grouped broadly by measurement type.

| **Measurement Type** | **Included Measurements** |
| --- | --- |
| Demographic or encounter overview quantities | Age, sex, time since first vitals measurement, incoming transfer from ICU |
| Vitals | Heart rate, respiration rate, systolic blood pressure, diastolic blood pressure, O2 saturation, temperature, AVPU |
| Flowsheet quantities | FiO2, urine output, Braden Scale assessments, BMI |
| Lab measurements | Albumin, bands (%), total bilirubin, blood urea nitrogen, carbon dioxide, chloride, creatinine, eosinophils (%), serum glucose, hemoglobin, international normalized ratio, lactate, lymphocytes (%), magnesium, mean corpuscular volume, monocytes (%), neutrophils (%), carbon dioxide arterial partial pressure, carbon dioxide venous partial pressure, arterial pH, venous pH, phosphate, platelet count, oxygen arterial partial pressure, potassium, partial thromboplastin time, red cell distribution width, serum glutamic oxaloacetic transaminase, sodium, white blood cells |

## Model Features

### Logistic Regression & Extreme Gradient Boosting

The LR and XGB models shared a common feature construction procedure, aside from their handling of missingness (as described in the main text). The first portion of the features for these two modeling approaches was the last available measurement for each of the measurement channels described in Table 1, with some minor modifications. First, Braden Scale measurements were processed into three features: an overall Braden Scale score, a Braden Activity score, and a Braden Nutrition score. Urine output was processed into a feature that assessed the total urine output over the 24 hours prior to the early warning score. Last, the chloride value was used (along with carbon dioxide and sodium) to create an ‘anion gap’ feature. Chloride was not used as a model feature, but carbon dioxide and sodium were retained as separate features. Collectively this resulted in 47 last-value-pulled-forward features. The LR and XGB features also included the following 20 temporal summary statistics: minimum systolic blood pressure, minimum diastolic blood pressure, minimum O2 saturation, maximum heart rate, maximum respiration rate, average FiO2, standard deviation of heart rate, standard deviation of respiration rate, standard deviation of systolic blood pressure, standard deviation of temperature, slope of heart rate, slope of temperature, slope of FiO2, change in blood urea nitrogen, change in creatinine, change in hemoglobin, change in phosphate, change in platelet count, change in potassium, and change in white blood cells. These quantities were calculated using measurements from the 24 hours prior to the eCART early warning flag (with a minimum 2 measurements, inclusive of measurements at the time of the elevated eCART score). Slope quantities refer to the slope between the first available measurement in the trend window and the final measured value (inclusive of measurements at eCART). Change quantities refer to the difference between the latest measurement and the penultimate measurement; change quantities were the only temporal summary statistics whose calculation was not limited to the 24 hours prior to the early warning score (i.e., could be calculated using older measurements, if necessary). If the minimum number of measurements was not available for calculation of any of the temporal summary statistics, then these values were left as missing (and, for LR, subsequently imputed using median values).

### Long Short-Term Memory

The LSTMs were trained with features constructed from each of the measurements available in Table 1, with minor modifications similar to LR and XGB. First, Braden Scale measurements were processed into three features: an overall Braden Scale score, a Braden Activity score, and a Braden Nutrition score. Urine output was processed into a feature that assessed the total amount of urine output over the prior 24 hours (relative to each resampled interval). Last, the chloride value was used (along with carbon dioxide and sodium) to create an ‘anion gap’ feature. Chloride was not used as a model feature, but carbon dioxide and sodium were retained as separate features. Collectively, this resulted in a total of 47 substantive features provided to the LSTMs. When imputation Booleans were used, a separate Boolean feature was added for each substantive feature indicating if the feature was imputed (1) or not (0) in the given resampling period. Note that features which were not expected to change or for which it did not make sense to indicate imputation status (age, sex, prior transfer from ICU, time since first vitals measurement, and 24-hour rolling sum of urine output) did not receive imputation Booleans (when applicable). In instances including imputation Booleans, the LSTMs were thus provided with 89 input features (47 substantive features and 42 imputation Booleans).

## Model Structure and Hyperparameters

### Logistic Regression

As noted in the main text, we implemented LR using Scikit learn [1], specifically using the ‘LogisticRegression’ class. We tested various forms of regularization (i.e., l1, l2, and elastic net), as well as regularization strength and class weighting during hyperparameter tuning. A complete set of hyperparameter ranges for LR can be found in Table 2 (see Section ‘Final Hyperparameter Ranges’). Note that the same hyperparameter ranges were used for both the individual LR models as well as the stacking ensemble. The best performing hyperparameters for each prediction task can be found in Tables 2 and 4 of Multimedia Appendix 2, for the individual LR models and the stacking ensembles, respectively. The ‘liblinear’ solver (max iteration count of 1000) was used for both l1 and l2 regularized models while the ‘saga’ solver (max iteration count of 10000) was used for elastic net regularized models. Any model parameter not specified here or in the ranges provided in Table 2 used the default values given for the LogisticRegression class in Scikit learn. LR hyperparameter tuning used the area under the ROC curve (‘roc_auc’) as the cross validation scoring function.

### Extreme Gradient Boosting

As noted in the main text, we implemented gradient-boosted tree models with Extreme Gradient Boosting (XGB) [2], specifically using the ‘XGBClassifier’ class. Tested hyperparameters for XGB hyperparameter tuning are provided in Table 3 (see Section ‘Final Hyperparameter Ranges’). The best performing hyperparameters for each prediction task can be found in Table 3 of Multimedia Appendix 2. Any model parameter not specified in Table 3 used the default parameters of the XGBClassifier class. XGB hyperparameter tuning used the area under the ROC curve (‘roc_auc’) as the cross validation scoring function.

### Long Short-Term Memory

As noted in the main text, we implemented long short-term memory models using PyTorch [3], specifically using the ‘LSTM’ class. Ranges for tested hyperparameters are provided in Table 1 in Multimedia Appendix 2. The best performing hyperparameters for each task are provided in Table 5 in Multimedia Appendix 2. When LSTMs included multiple hidden layers, all layers contained the same number of hidden units. If dropout was used, it operated only between hidden layers, not between the input features and the first hidden layer. During initial validation, we also explored the use of layering an additional feedforward neural network (with configurable size) on the output of the LSTM, but we found that it did not improve performance and thus did not include it in the final set of hyperparameters. For all testing, we used an encounter batch size of 32, PyTorch’s built-in binary cross entropy function as the model’s loss function (‘BCEWithLogitsLoss’), and the Adam optimizer [4] (with learning rate and weight decay tuned as part of hyperparameter tuning). During initial validation we also identified that class weighting appeared to be beneficial and thus used class weighting for all prediction tasks to reduce the size of the hyperparameter tuning space. Also note that the hyperparameter tuning process described in the main text used a patience parameter of 10 epochs (i.e., early stopping was triggered if no improvement was recorded in 10 epochs). Particularly for prediction tasks with more severe class imbalances, we noticed high volatility in the AUC performance after only a few epochs, so we chose to begin monitoring for early stopping after 5 epochs (for all prediction tasks) to ensure more stable hyperparameter performance estimates.

### Initial Validation Procedures

Initial work used an internal validation process to steer broad choices in tested hyperparameters, particularly for the tested LSTMs. An internal validation set was constructed by randomly selecting 150 encounters from each of the three training sites. This led to an internal training set of 1408 encounters and an internal validation set of 450 encounters (i.e., the external site test set was not used in any way during internal validation). We used a similar cross-validation process on the internal training set (as compared to that presented in the main text) to perform initial model testing with a coarsely defined range of hyperparameters. After confirming that the models performed well on the internal validation set and based on the best performing hyperparameters found during this internal cross-validation process, we constructed the final hyperparameter ranges used for each prediction task. Given the large number of available hyperparameters and the computational expense of LSTM hyperparameter tuning, this decision allowed for the Bayesian optimization-based hyperparameter tuning to focus its exploration on a more appropriate range of values for each prediction task. The final ranges used for training the models presented in the main text are provided in the subsequent sections, including the best-performing hyperparameters used to calculate performance results.

### Final Hyperparameter Ranges

Tables 2 and 3 contain the hyperparameter ranges for LR and XGB used during final hyperparameter tuning. Due to space constraints, the corresponding hyperparameter ranges for the LSTM models can be found in Table 1 in Multimedia Appendix 2. Any hyperparameters not noted took the default values associated with each package.

**Table 2.** Hyperparameter value ranges used for logistic regression hyperparameter tuning (via grid search). The same grid was used for all prediction tasks. Note that these hyperparameters were used both for the individual logistic regression models as well as the stacking ensemble meta-learner logistic regression model.

| **Hyperparameter** | **Description** | **Grid Values** |
| --- | --- | --- |
| ‘penalty’ | Type of regularization used in the model | (‘l1’, ’l2’, ‘elasticnet’) |
| ‘C’ | Inverse of regularization strength | (0.001, 0.01, 0.1, 1, 10, 100, 1000) |
| ‘l1_ratio’ | (Elastic net only) The ratio between l1 and l2 regularization. | (0.1, 0.3, 0.5, 0.7, 0.9) |
| ‘class_weight’ | Weighting used for each class label (note that ‘balanced’ implies a weighting inversely proportional to the label frequencies in the training set, while None implies a weight of one for all classes) | (‘balanced’, None) |

**Table 3.** Hyperparameter value ranges used for XGBoost hyperparameter tuning (via grid search). The same grid was used for all prediction tasks.

| **Hyperparameter** | **Description** | **Grid Values** |
| --- | --- | --- |
| ‘n_estimators’ | Number of gradient boosted trees | (20, 40, 60, …, 380, 400) |
| ‘eta’ | Learning rate | (0.1) |
| ‘colsample_bytree’ | Subsample ratio of columns for each depth level | (0.5, 0.75, 1) |
| ‘subsample’ | Subsample ratio of training instances | (0.5, 0.75, 1) |
| ‘max_depth’ | Maximum tree depth | (1, 2, 3, 4, 5) |
| ‘min_child_weight’ | Minimum sum of instance weight (hessian) needed in a child node | (1, 2, 3, 4, 5) |
| ‘scale_pos_weight’ | Class weighting (calculated identically to the logistic regression case using label frequencies in the training set) | (True, False)^[[1]](#footnote-1)^ |

### Best Performing Hyperparameters

Due to space constraints, the best performing hyperparameters for each model (including the stacking ensemble) and prediction task can be found in Tables 2-5 in Multimedia Appendix 2.

## Treatment Timing

Table 4 provides the complete counts of encounters at each studied health system, including the chart-reviewed lifesaving treatment labels.

**Table 4.** Number of positive labels for each of the studied health systems. Note that individual encounters could receive multiple lifesaving treatment labels, meaning that total counts of positive cases across treatments do not necessarily sum to the health systems’ encounter counts.

| **Treatment** | **University of Chicago Medical Center (n=483)** | **University of Wisconsin-Madison Hospital (n=656)** | **NorthShore University HealthSystem (n=719)** | **Loyola University Medical Center (n=622)** | **Total (n=2480)** |
| --- | --- | --- | --- | --- | --- |
| Antimicrobial | 162 | 344 | 340 | 300 | 1146 |
| Fluid Bolus | 125 | 233 | 244 | 231 | 833 |
| Antiarrhythmic | 106 | 92 | 154 | 111 | 463 |
| Diuretic | 71 | 91 | 90 | 93 | 345 |
| Inh. Bronch. | 43 | 51 | 105 | 79 | 278 |
| Transfusion | 39 | 43 | 49 | 60 | 191 |
| Invasive Vent. | 61 | 72 | 18 | 53 | 204 |
| Vasoactive | 55 | 52 | 26 | 49 | 182 |
| Anticoagulant | 56 | 30 | 57 | 36 | 179 |
| Steroid | 36 | 63 | 72 | 29 | 200 |

To supplement the summaries of treatment initiation timing provided in the main text, Figure 1 displays kernel density estimates for when individuals first received each treatment within the 48-hour window on either side of their high eCART warning flag.

**Figure 1.** Kernel density estimates for the time of first treatment relative to time of high eCART score. Note that this kernel density estimate considers only the subset of encounters with treatments provided within a 48-hour window on either side of the eCART flag. Individuals may have received treatment outside this window or multiple times within this window, but here ‘first treatment’ refers to the first time the individual is treated within this window. The percentage provided alongside each treatment indicates the fraction of encounters included in the kernel density estimate (out of all encounters labeled with that particular lifesaving treatment). This means that any encounter not included in this kernel density estimate did not contain administration of the labeled lifesaving treatment within 48 hours on either side of the early warning flag. The time of the eCART flag is assigned a value of zero, so negative values of ‘treatment delta’ correspond to instances where treatment is started prior to the eCART flag while positive values indicate cases where treatment began after the eCART flag. As mentioned in the main text, invasive ventilation is not included as there was no reliable EHR measurement indicating when treatment began.

## Calibration Curves

To supplement the pooled calibration curves provided in the main text, Figure 2 provides calibration curves for each model, separated by prediction task.

**Figure 2.** Calibration curves for the tested algorithms, with each panel representing a separate prediction task (i.e., separate curves for each model type are calculated for each treatment type). Note that the relatively small sample size and (in some cases) severe class imbalance makes calibration assessment difficult.

## Performance Metric Confidence Intervals

### Area under the ROC Curve

Table 5 provides median AUC values and 95% confidence intervals for each model and prediction task.

**Table 5.** 95% confidence intervals for AUC calculated using 10,000 bootstraps (sampling with replacement). Each bootstrap resampled values until reaching 70% of the original dataset’s size. Percentile values are provided in the form (2.5%, 50%, 97.5%).

| **Treatment** | **LSTM Multi-label** | **LSTM Single-label** | **Logistic Regression** | **XGBoost** | **Ensemble** |
| --- | --- | --- | --- | --- | --- |
| Antimicrobial | (0.760, 0.774, 0.788) | (0.770, 0.783, 0.797) | (0.762, 0.776, 0.789) | (0.783, 0.797, 0.809) | (0.797, 0.810, 0.822) |
| Fluid Bolus | (0.695, 0.710, 0.726) | (0.699, 0.715, 0.731) | (0.705, 0.722, 0.737) | (0.709, 0.725, 0.741) | (0.722, 0.738, 0.754) |
| Antiarrhythmic | (0.833, 0.852, 0.870) | (0.854, 0.869, 0.884) | (0.864, 0.879, 0.894) | (0.836, 0.854, 0.872) | (0.861, 0.877, 0.892) |
| Diuretic | (0.670, 0.692, 0.714) | (0.644, 0.668, 0.691) | (0.675, 0.699, 0.722) | (0.704, 0.725, 0.747) | (0.700, 0.722, 0.744) |
| Inh. Bronch. | (0.756, 0.779, 0.800) | (0.753, 0.775, 0.796) | (0.761, 0.783, 0.803) | (0.840, 0.856, 0.871) | (0.813, 0.833, 0.852) |
| Transfusion | (0.783, 0.803, 0.822) | (0.821, 0.838, 0.855) | (0.798, 0.818, 0.838) | (0.803, 0.822, 0.841) | (0.817, 0.836, 0.854) |
| Invasive vent. | (0.641, 0.668, 0.695) | (0.584, 0.616, 0.647) | (0.617, 0.648, 0.678) | (0.692, 0.718, 0.743) | (0.678, 0.704, 0.730) |
| Vasoactive | (0.632, 0.661, 0.689) | (0.589, 0.623, 0.657) | (0.665, 0.696, 0.726) | (0.634, 0.663, 0.693) | (0.672, 0.701, 0.728) |
| Anticoagulant | (0.673, 0.709, 0.744) | (0.515, 0.548, 0.581) | (0.663, 0.701, 0.737) | (0.623, 0.661, 0.695) | (0.645, 0.682, 0.717) |
| Steroid | (0.698, 0.734, 0.768) | (0.720, 0.753, 0.784) | (0.653, 0.689, 0.725) | (0.680, 0.722, 0.760) | (0.738, 0.770, 0.799) |

### Brier Scores

Table 6 provides median Brier score values and 95% confidence intervals for each model and prediction task.

**Table 6.** 95% Confidence intervals for Brier Scores calculated using 10,000 bootstraps (sampling with replacement). Each bootstrap resampled values until reaching 70% of the original dataset’s size. Percentile values are provided in the form (2.5%, 50%, 97.5%).

| **Treatment** | **LSTM Multi-label** | **LSTM Single-label** | **Logistic Regression** | **XGBoost** | **Ensemble** |
| --- | --- | --- | --- | --- | --- |
| Antimicrobial | (0.201, 0.206, 0.210) | (0.191, 0.197, 0.202) | (0.190, 0.195, 0.201) | (0.179, 0.185, 0.190) | (0.175, 0.180, 0.186) |
| Fluid Bolus | (0.216, 0.220, 0.224) | (0.199, 0.205, 0.211) | (0.196, 0.202, 0.208) | (0.202, 0.207, 0.212) | (0.192, 0.198, 0.205) |
| Antiarrhythmic | (0.155, 0.161, 0.166) | (0.120, 0.125, 0.131) | (0.083, 0.089, 0.095) | (0.083, 0.089, 0.095) | (0.078, 0.084, 0.090) |
| Diuretic | (0.166, 0.170, 0.174) | (0.209, 0.215, 0.221) | (0.171, 0.177, 0.182) | (0.108, 0.116, 0.123) | (0.174, 0.179, 0.184) |
| Inh. Bronch. | (0.184, 0.189, 0.194) | (0.145, 0.150, 0.155) | (0.093, 0.100, 0.108) | (0.088, 0.094, 0.102) | (0.089, 0.096, 0.104) |
| Transfusion | (0.198, 0.204, 0.210) | (0.163, 0.169, 0.175) | (0.205, 0.209, 0.214) | (0.067, 0.073, 0.079) | (0.066, 0.071, 0.077) |
| Invasive vent. | (0.287, 0.293, 0.298) | (0.299, 0.306, 0.313) | (0.071, 0.077, 0.084) | (0.069, 0.075, 0.081) | (0.248, 0.254, 0.259) |
| Vasoactive | (0.211, 0.217, 0.223) | (0.179, 0.185, 0.191) | (0.222, 0.225, 0.228) | (0.068, 0.074, 0.081) | (0.065, 0.072, 0.078) |
| Anticoagulant | (0.204, 0.209, 0.213) | (0.312, 0.320, 0.327) | (0.222, 0.225, 0.227) | (0.149, 0.153, 0.158) | (0.050, 0.055, 0.060) |
| Steroid | (0.261, 0.267, 0.272) | (0.132, 0.136, 0.141) | (0.041, 0.045, 0.050) | (0.045, 0.050, 0.056) | (0.224, 0.227, 0.229) |

## Feature Importance

### SHAP Analysis of Steroid Treatments

The main text noted that steroid prediction displayed a counterintuitive increase in predictive performance when evaluated on untreated patients as compared to the complete test set. We noted this trend for all models and take XGB as an illustrative example. Figure 3 provides an assessment of global feature importance for XGB predictions made on the complete test set, assessed by the average absolute SHAP value for each feature. Figure 4 displays boxplots of the covariate distributions for the 10 most important features (as shown in Figure 3) for the complete test set population. Figure 5 further shows the covariate distributions for the same features but restricted to the subpopulation of the test set that was positively labeled (i.e., those encounters where steroids were indicated as a lifesaving treatment by manual chart review). These data did not suggest a clear explanation for why discriminative performance would improve on the untreated patient population and is a phenomenon we plan to evaluate in future work.

**Figure 3.** The 10 most important covariates for XGB steroid prediction, as assessed by SHAP values on the complete test site cohort. Values are given in descending order of mean |SHAP| value, averaged over the test set encounters.

**Figure 4.** Boxplots of the distributions of the 10 most important covariates for steroid prediction, identified by SHAP analysis, for the complete test set population (including both positively and negatively labeled encounters). ‘All’ indicates that the boxplot shows the covariate distribution for all encounters. ‘Untreated’ indicates that the boxplot shows the covariate distribution for the subset of encounters where the patient did not receive treatment in the 48-hour window prior to the studied eCART early warning flag. Note that features have been normalized to allow presentation on a common axis.

**Figure 5.** Boxplots of the distributions of the 10 most important covariates for steroid prediction, identified by SHAP analysis, for the subset of the test set patient population with positive steroid treatment labels. ‘All’ indicates that the boxplot shows the covariate distribution for all such encounters. ‘Untreated’ indicates that the boxplot shows the covariate distribution for the further subset of encounters where the patient did not receive treatment in the 48-hour window prior to the studied eCART early warning flag. Note that features have been normalized to allow presentation on a common axis.

### SHAP Analysis of Anticoagulant Treatments

The main text presents a case study of anticoagulant prediction to illustrate a possible benefit of time-series approaches for predicting the need for certain lifesaving treatments. Figure 6 provides an assessment of global feature importance for XGB anticoagulant predictions made on the test set, assessed by the average absolute SHAP value for each feature. In this case, we note that the 3 most important features are temporal summary statistics, with additional temporal summary statistics appearing in the list of the 11 most important features (i.e., standard deviation of respiration rate and maximum heart rate).

**Figure 6.** The 11 most important covariates for XGB anticoagulant prediction, as assessed by SHAP values on the complete test site cohort. Values are given in descending order of mean |SHAP| value, averaged over the dataset.

### Additional SHAP Analysis for Common Treatments

As noted in the main text, we provide similar XGB global SHAP analysis for the 3 most common treatments (antimicrobials, fluid boluses, and antiarrhythmics) to demonstrate that the most important model features tended to align well with clinical intuition. Figures 7-9 provide global SHAP results for predictions made on the test set, assessed by the average absolute SHAP value for each feature, for antimicrobials, fluid boluses, and antiarrhythmics, respectively.

**Figure 7.** The 10 most important covariates for XGB antimicrobial prediction, as assessed by SHAP values on the complete test site cohort. Values are given in descending order of mean |SHAP| value, averaged over the dataset.

**Figure 8.** The 10 most important covariates for XGB fluid bolus prediction, as assessed by SHAP values on the complete test site cohort. Values are given in descending order of mean |SHAP| value, averaged over the dataset.

**Figure 9.** The 10 most important covariates for XGB antiarrhythmic prediction, as assessed by SHAP values on the complete test site cohort. Values are given in descending order of mean |SHAP| value, averaged over the dataset.

# References

1. Pedregosa F, Varoquaux G, Gramfort A, Michel V, Thirion B, Grisel O, Blondel M, Prettenhofer P, Weiss R, Dubourg V, Vanderplas J, Passos A, Cournapeau D, Brucher M, Perrot M, Duchesnay É. Scikit-learn: Machine Learning in Python. J Mach Learn Res 2011;12(85):2825–2830.

2. Chen T, Guestrin C. XGBoost: A Scalable Tree Boosting System. Proc 22nd ACM SIGKDD Int Conf Knowl Discov Data Min San Francisco California USA: ACM; 2016. p. 785–794. doi: 10.1145/2939672.2939785

3. Paszke A, Gross S, Massa F, Lerer A, Bradbury J, Chanan G, Killeen T, Lin Z, Gimelshein N, Antiga L, Desmaison A, Kopf A, Yang E, DeVito Z, Raison M, Tejani A, Chilamkurthy S, Steiner B, Fang L, Bai J, Chintala S. PyTorch: An Imperative Style, High-Performance Deep Learning Library. Adv Neural Inf Process Syst Curran Associates, Inc.; 2019. Available from: https://proceedings.neurips.cc/paper/2019/hash/bdbca288fee7f92f2bfa9f7012727740-Abstract.html [accessed Dec 21, 2022]

4. Kingma DP. Adam: A method for stochastic optimization. ArXiv Prepr ArXiv14126980 2014;

1. Note that the grid values were not truly ‘True’ and ‘False’ as XGBoost takes the particular class weighting calculation as the input value for this parameter. As this varied by prediction task, we represent it here using these binary choices. In the case of ‘False,’ uniform weighting is applied, as in the ‘None’ case for logistic regression’s ‘class_weight’ parameter. [↑](#footnote-ref-1)
