# Supplementary material for: Treatment Recommendations for Clinical Deterioration on the Wards: Development and Validation of Machine Learning Models
Source: JMIR AI. 2026 Jan 16;5:e81642. doi: 10.2196/81642 (PMC12810948; doi:10.2196/81642)
Supplement: Multimedia Appendix 3 [file ai-v5-e81642-s003.docx]

# Multimedia Appendix 2

This appendix houses oversized tables requiring landscape orientation, as referenced in Multimedia Appendix 1.

Table 1 describes the hyperparameter ranges evaluated for the various LSTM models. Tables 2, 3, 4, and 5 contain the best performing hyperparameters for each prediction task for the individual LR models, the XGB models, the stacking ensemble models, and the LSTM models, respectively.

**Table 1.** Hyperparameter value ranges used for LSTM hyperparameter tuning (via Bayesian optimization). The value ranges varied by task and are displayed accordingly. Parentheses denote a collection of discrete values that could be selected during tuning while brackets denote inclusive intervals, given by [lower bound, upper bound], that could be explored during tuning via Bayesian optimization.

**Table 2.** The best performing hyperparameters for the LR models, separated by prediction task. ‘N/A’ indicates a parameter is not applicable (i.e., l1_ratio is only valid when elastic net regularization is used).

**Table 3.** The best performing hyperparameters for the XGB models separated by prediction task.

**Table 4.** The best performing hyperparameters for the stacking ensemble models (logistic regression), separated by prediction task. ‘N/A’ indicates a parameter is not applicable (i.e., l1_ratio is only valid when elastic net regularization is used).

**Table 5.** The best performing hyperparameters for the LSTMs, separated by prediction task. Note that the multi-label model made predictions for all outcomes using the same model and thus has a single set of hyperparameters for all tasks (and is thus represented as an additional column here).
