# Supplementary material for: Treatment Recommendations for Clinical Deterioration on the Wards: Development and Validation of Machine Learning Models
Source: JMIR AI. 2026 Jan 16;5:e81642. doi: 10.2196/81642 (PMC12810948; doi:10.2196/81642)
Supplement: Checklist 1 [file ai-v5-e81642-s004.docx]

**Consolidated reporting guidelines for prognostic and diagnostic machine learning modeling studies**

The following is the reporting checklist. A response should indicate whether the particular item is documented in the study. If the response to an item is Y then the location in the article should be provided (e.g., section number), and if the response is N or NA then some reasoning should be provided.

| **#** | **Item** | **Y** | **N** | **NA** | **Location / Reasoning** |
| --- | --- | --- | --- | --- | --- |
| **Study Details** | | | | | |
| 1.1 | *The medical/clinical task of interest* | X |  |  | The prediction task and corresponding motivation is described in detail in the Introduction. Additional details for the specifics of the prediction task are described in the Methods. |
| 1.2 | *The research question* | X |  |  | This is described in the Introduction. |
| 1.3 | *Current medical/clinical practice* | X |  |  | We provide an overview of existing practice in the Introduction. |
| 1.4 | *The known predictors and confounders to what is being predicted / diagnosed* | X |  |  | We provide an overview in the Introduction and revisit these points on a more granular basis in the Discussion subsections. |
| 1.5 | *The overall study design* | X |  |  | An overview is provided in the Introduction subsection titled Contribution and a detailed design is given in the Methods. |
| 1.6 | *The medical institutional setting(s)* | X |  |  | This is described in the Methods (see Study Cohort). |
| 1.7 | *The target patient population* | X |  |  | This is described in the Introduction and Methods (see Study Cohort). |
| 1.8 | *The intended use of the ML model* | X |  |  | This is described in the Introduction and Methods (see Study Cohort). |
| 1.9 | *Existing model performance benchmarks for this task* | X |  |  | We note in the Introduction and Discussion that models of this kind (prediction of clinical deterioration interventions trained using gold-standard chart reviewed data from multiple sites) do not yet exist in the literature. |
| 1.10 | *Ethical and other regulatory approvals obtained* | X |  |  | We provide all relevant IRB approvals in the Methods (see Study Cohort and Ethical Considerations). |
| **The Data** | | | | | |
| 2.1 | *Inclusion / exclusion criteria for the patient cohort* | X |  |  | These procedures are described in the Methods (see Study Cohort and Chart Review). |
| 2.2 | *Methods of data collection* | X |  |  | See Methods (Study Cohort, Patient Measurements, and Chart Review). Additional information regarding collected data content is available in Multimedia Appendix 1. |
| 2.3 | *Bias introduced due to the method of data collection used* | X |  |  | We note a few limitations (e.g., the use of data from regionally similar hospitals potentially affecting the generalization of our findings) in the Discussion (see Limitations). |
| 2.4 | *Data characteristics* | X |  |  | These are described in detail in the Methods (see Measures) as well as in Multimedia Appendix 1. |
| 2.5 | *Methods of data transformations and preprocessing applied* | X |  |  | See Methods (Feature Engineering) as well as Multimedia Appendix 1. |
| 2.6 | *Known quality issues with the data* | X |  |  | We note such issues in the Methods (see Study Cohort and Measures) as well as in Multimedia Appendix 1. |
| 2.7 | *Sample size calculation* |  |  | X | Size of the chart reviewed cohort was fixed by the previously cited study. |
| 2.8 | *Data Availability* | X |  |  | We provide a data availability statement in the section Data Availability. |
| **Methodology** | | | | | |
| 3.1 | *Strategies for handling missing data* | X |  |  | Provided in the Methods (see Feature Engineering). |
| 3.2 | *Strategies for addressing class imbalance* | X |  |  | Provided in the Methods (see Model Training). |
| 3.3 | *Strategies for reducing dimensionality of data* | X |  |  | Provided in the Methods (see Feature Engineering, e.g., for the construction of temporal summary features). |
| 3.4 | *Strategies for handling outliers* |  |  | X | Data were not assessed to be/not be outliers. No model training procedures depended on handling outliers. |
| 3.5 | *Strategies for data augmentation* |  |  | X | Data augmentation was not used. Our data procedures are clearly defined in the Methods. |
| 3.6 | *Strategies for model pre-training* |  |  | X | Model pre-training was not used (at least not in the context of a foundation-type model). Models were trained directly on the clinical data described in the Methods. |
| 3.7 | *The rationale for selecting the machine learning algorithm* | X |  |  | See Introduction and Methods. An expressed purpose of this study was evaluating which types of models perform well for clinical deterioration treatment prediction, so we chose to evaluate a variety of different model types. |
| 3.8 | *The method of evaluating model performance during training* | X |  |  | See Methods (Model Training and Evaluation Criteria). |
| 3.9 | *The method used for hyperparameter tuning* | X |  |  | See Methods (Model Training). |
| 3.10 | *Model’s output adjustments* | X |  |  | See Methods (Model Training). Largely, this was not relevant except in the context of training the stacking ensemble using individual model outputs. We did not perform other adjustments (e.g., calibration adjustments) as part of this work. |
| **Evaluation** | | | | | |
| 4.1 | *Performance metrics used to evaluate the model* | X |  |  | See Methods (Evaluation Criteria). |
| 4.2 | *The cost or consequence of errors* |  |  | X | See Methods (Evaluation Criteria). We note that discriminative performance is the primary metric of our work. We are explicit in the main text that we do not address practical misclassification costs as part of this study. |
| 4.3 | *The results of internal validation* | X |  |  | These are summarized in the Methods (Model Training). |
| 4.4 | *The final model hyperparameters* | X |  |  | We summarize procedures in the Methods and provide hyperparameter values in Multimedia Appendices 1 and 2. |
| 4.5 | *Model evaluation on an external dataset* | X |  |  | See Results (models were trained on data from 3 health systems and evaluated on data from a separate health system). |
| 4.6 | *Characteristics relevant for detecting data shift and drift* |  |  | X | This was not in the scope of this study. |
| **Explainability and Transparency** | | | | | |
| 5.1 | *The most important features and how they relate to the outcome(s)* | X |  |  | See Discussion for a detailed breakdown of the results (e.g., by prediction task, by model type, etc.). Some of the case studies in the Discussion specifically address feature importance, with additional details provided in Multimedia Appendix 1. |
| 5.2 | *Plausibility of model outputs* | X |  |  | See Methods. Model inputs are ubiquitous EHR data fields. |
| 5.3 | *Interpretation of model's results by an end-user* | X |  |  | See Discussion. |
